# A reference genetic map of *C. clementina* hort. ex Tan.; citrus evolution inferences from comparative mapping

**DOI:** 10.1186/1471-2164-13-593

**Published:** 2012-11-05

**Authors:** Patrick Ollitrault, Javier Terol, Chunxian Chen, Claire T Federici, Samia Lotfy, Isabelle Hippolyte, Frédérique Ollitrault, Aurélie Bérard, Aurélie Chauveau, Jose Cuenca, Gilles Costantino, Yildiz Kacar, Lisa Mu, Andres Garcia-Lor, Yann Froelicher, Pablo Aleza, Anne Boland, Claire Billot, Luis Navarro, François Luro, Mikeal L Roose, Frederick G Gmitter, Manuel Talon, Dominique Brunel

**Affiliations:** 1 CIRAD, UMR AGAP, F-34398 Montpellier, France; 2 IVIA, Centro Proteccion Vegetal y Biotechnologia, Ctra. Moncada-Náquera Km 4.5, 46113 Moncada, Valencia, Spain; 3 IVIA, Centro de Genomica, Apartado Oficial, 46113, Moncada, Valencia, Spain; 4 Citrus Research and Education Center, University of Florida, Lake Alfred, FL, 33850, USA; 5 Department of Botany and Plant Sciences, University of California, Riverside, CA, 92521, USA; 6 Institut National de la Recherche Agronomique, BP, 293, 14 000, Kénitra, Morocco; 7 INRA, UR EPGV, 2 rue Gaston Cremieux, 91057, Evry, France; 8 INRA, UR GEQA, San Giuliano, 20230, San Nicolao, France; 9 Department of Horticulture, Faculty of Agriculture, University of Çukurova, 01330, Adana, Turkey; 10 CNG, CEA/DSV/Institut de Génomique, 2 rue Gaston Cremieux, 91057, Evry, France

**Keywords:** *C. clementina*, *C. sinensis*, *C. maxima*, SSRs, SNPs, Indels, Genetic maps

## Abstract

**Background:**

Most modern citrus cultivars have an interspecific origin. As a foundational step towards deciphering the interspecific genome structures, a reference whole genome sequence was produced by the International Citrus Genome Consortium from a haploid derived from Clementine mandarin. The availability of a saturated genetic map of Clementine was identified as an essential prerequisite to assist the whole genome sequence assembly. Clementine is believed to be a ‘Mediterranean’ mandarin × sweet orange hybrid, and sweet orange likely arose from interspecific hybridizations between mandarin and pummelo gene pools. The primary goals of the present study were to establish a Clementine reference map using codominant markers, and to perform comparative mapping of pummelo, sweet orange, and Clementine.

**Results:**

Five parental genetic maps were established from three segregating populations, which were genotyped with Single Nucleotide Polymorphism (SNP), Simple Sequence Repeats (SSR) and Insertion-Deletion (Indel) markers. An initial medium density reference map (961 markers for 1084.1 cM) of the Clementine was established by combining male and female Clementine segregation data. This Clementine map was compared with two pummelo maps and a sweet orange map. The linear order of markers was highly conserved in the different species. However, significant differences in map size were observed, which suggests a variation in the recombination rates. Skewed segregations were much higher in the male than female Clementine mapping data. The mapping data confirmed that Clementine arose from hybridization between ‘Mediterranean’ mandarin and sweet orange. The results identified nine recombination break points for the sweet orange gamete that contributed to the Clementine genome.

**Conclusions:**

A reference genetic map of citrus, used to facilitate the chromosome assembly of the first citrus reference genome sequence, was established. The high conservation of marker order observed at the interspecific level should allow reasonable inferences of most citrus genome sequences by mapping next-generation sequencing (NGS) data in the reference genome sequence. The genome of the haploid Clementine used to establish the citrus reference genome sequence appears to have been inherited primarily from the ‘Mediterranean’ mandarin. The high frequency of skewed allelic segregations in the male Clementine data underline the probable extent of deviation from Mendelian segregation for characters controlled by heterozygous loci in male parents.

## Background

Citrus fruits were domesticated in South East Asia several thousand years ago and subsequently spread throughout the world. Today, the area of citrus cultivation is primarily found between the latitudes of 40°N and 40°S, and global citrus production has reached 122 M tonnes [1]. The production of sweet orange, the leading varietal type, approaches close to 69 M tonnes [1]. Small citrus fruits (mandarin-like) are preponderant in China and very important in the Mediterranean Basin where Clementine is the main cultivar.

Despite controversial *Citrus* classifications, most authors now agree on the origin of cultivated citrus species. Scora [2] and Barrett and Rhodes [3] were the first to suggest that three primary *Citrus* species *(C. medica* L. – citrons, *C. reticulata* Blanco – mandarins, and *C. maxima* L. Osbeck – pummelos) were the ancestors of most cultivated citrus. The differentiation between these sexually compatible taxa can be explained via the foundation effect in three geographic zones and by an initial allopatric evolution [2,4]. Other cultivated species (referred to hereafter as secondary species) such as *C. aurantium* L. (sour orange), *C. sinensis* (L.) Osb. (sweet orange), *C. paradisi* Macf. (grapefruit), *C. clementina* hort. Ex Tan. (Clementine) and *C. limon* Osb. (lemon) originated later through hybridization and a limited number of sexual recombination events among the basic taxa. Molecular marker studies [5-8] generally support the role of these three taxa as ancestors of cultivated *Citrus*. Furthermore, some of these studies [8-10] highlighted the probable contribution of a fourth taxon, *C. micrantha* Wester, as the ancestor of some limes *C. aurantifolia* (Christm.) Swingle].

In general, *Citrus* species are diploid with a basic chromosome number *x* = 9 [11]. *Citrus* species have small genomes. While estimating citrus genome size by flow cytometry, Ollitrault et al. [12] found significant genome size variation between citrus species. The largest and smallest genomes were *C. medica* (average value of 398 Mb/haploid genome) and *C. reticulata* (average value of 360 Mb/haploid genome), respectively. *C. maxima* had an intermediate genome size, with an average value of 383 Mb/haploid genome. Interestingly, the secondary species presented intermediate values between their putative ancestral parental taxa, *C. sinensis* (370 Mb), *C. aurantium* (368 Mb), *C. paradisi*, (381 Mb) and *C. limon* (380 Mb) per haploid genome.

**Figure 1:**
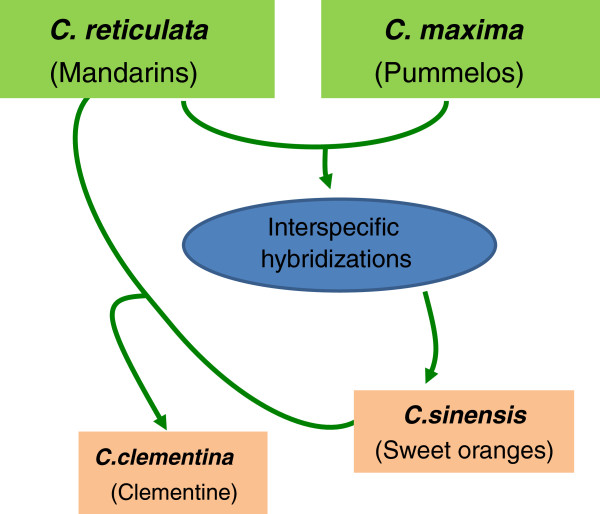
**Assumed parentage relationships between *C. reticulata*, *C. maxima*, *C. sinensis *and *C. clementina.*** From Ollitrault et al. [8].

As mentioned previously, most modern cultivars have an interspecific origin and their genomes can be considered mosaics of large DNA fragments inherited from the basic taxa [7]. These cultivars are generally highly heterozygous [6,7]. The *C. maxima* and *C. reticulata* gene pools contributed to the genesis of most of the economically important species and cultivars including sweet and sour oranges, grapefruits, tangors (mandarin × sweet orange hybrids), tangelos (mandarin × grapefruit hybrids) and lemons [6,7,9]. Barkley et al. and Garcia-Lor et al. [10,11] estimated the relative contributions of primary species to modern cultivars. Some discrepancies have been observed between these studies, and the detailed interspecific genome organization of cultivated secondary species and modern cultivars is still largely unknown. As a foundational step towards deciphering the phylogenetic structures of citrus genomes and the molecular bases of phenotypic variation, a reference whole genome sequence of a haploid derived from Clementine was produced and is currently being revised by the International Citrus Genome Consortium (ICGC) [13,14]. The Clementine mandarin is an interspecific hybrid that was selected one century ago in Algeria by Father Clement as a chance offspring among seedlings of the ‘Mediterranean’ mandarin (*C. reticulata*) [15]. Since that time, the Clementine has been vegetatively propagated by grafting. In a recent large SNP diversity survey, Ollitrault et al. [8] confirmed that the Clementine is a ‘Mediterranean’ mandarin × sweet orange hybrid (tangor). This conclusion is in agreement with the hypothesis of Deng et al. and Nicolosi et al. [9,16] The supposed parental relationships between Clementine, sweet orange, pummelo and mandarin are summarized in Figure 1. The Clementine genome size is estimated to be 367 Mb/haploid genome [12].

The ICGC identified the construction of a saturated genetic map of Clementine as an essential prerequisite to improve the sequence assembly of the haploid Clementine reference genome. Compared with other crops, genetic mapping in citrus is relatively less well developed. The partial genetic maps built with codominant markers (primarily SSRs) [17-19] encompass around 150 markers, while maps based on dominant markers such as AFLPs, [20] SRAPs, ISSRs, and RAPDs [21] include slightly more than 200 markers. Moreover, few of the mapped markers have been published in GenBank (or other public nucleotide databases). Within the last 15 years, the citrus community developed Simple Sequence Repeat (SSR) markers with reference sequences that were deposited in public databases. While a limited number of SSR markers were obtained from genomic libraries [6,22-24], the implementation of large EST databases allowed the development of many more SSR markers [25,26], and additional markers have been developed from Clementine BACs end sequencing (BES; [27-29]). From the same Clementine BES database, Ollitrault et al. [30] developed 33 Indel markers to contribute to Clementine genetic mapping. Despite these international efforts, the number of available heterozygous SSRs and Indels in Clementine was still insufficient to establish a saturated Clementine genetic map. SNP markers are well adapted for high throughput methods for marker saturation. Ollitrault et al. [8] took advantage of the Clementine BES database [27] to identify SNPs heterozygous in Clementine, and a GoldenGate SNPs array was developed. Interestingly, 63% of the validated SNP markers were heterozygous in the sweet orange. Therefore, these SNPs can be used for comparative mapping between the Clementine and sweet orange.

The primary goals of the present study were: (i) to establish a saturated reference map of Clementine using codominant markers with sequences available in public databases; (ii) to perform comparative mapping between sweet orange, pummelo and Clementine; and (iii) to localize the crossover events that produced the sweet orange gamete that contributed to the Clementine genome, and those involved in the gamete formation that gave rise to the haploid Clementine [13] used for the citrus reference whole genome sequence [14]. The clementine reference map and the pummelo map were established from two interspecific hybrid populations (‘Chandler’ pummelo × ‘Nules’ Clementine – CP × NC (156 hybrids) and ‘Nules’ Clementine × ‘Pink’ pummelo – NC × PP, (140 hybrids)) with 1166 codominant markers. The sweet orange map anchored with the Clementine map was established by genotyping 582 segregating SNP markers from 147 progeny from crosses between sweet orange and trifoliate orange (SO × TO). This study also yielded information regarding the magnitude and distribution of segregation distortion within the different crosses.

## Results

### Polymorphism and allele calls for the SNP markers

For all SNPs, genotyping was visually confirmed, taking advantage of the distribution of the segregating progenies relative to the parental positions. This observation was conducted individually for each plate of 96 genotypes. Plate/marker combinations with unclear clustering of genotypes were removed from the analysis. No differences were found between the different sweet orange parents or between the trifoliate orange parents of the SO × TO progenies. Therefore, all individuals resulting from the different crosses were considered as single family. For the selected data, the markers were assigned to different categories based on the observed segregations, the detection of null alleles and, finally, the type of segregation assumed according to the JoinMap nomenclature (Tables 1 and 2).

**Table 1:** Join map codification for the different allelic configurations encountered for SNP markers

	**AA**	**AB**	**BB**	**A0**	**B0**	**00**
**AA**	–	lmxll	–	lmxll	lmxll	–
**AB**	nnxnp	hkxhk	nnxnp	nnxnp	nnxnp	nnxnp
**BB**	–	lmxll	–	lmxll	lmxll	–
**A0**	nnxnp	lmxll	nnxnp	NO	NO	nnxnp
**B0**	nnxnp	lmxll	nnxnp	NO	NO	nnxnp
**00**	–	lmxll	–	lmxll	lmxll	–

NO: Non observed configuration.

**Table 2:** Segregation types observed for the different parents and progenies

			**SSRs**	**Indels**	**SNPs**	**Total**
Null allele	Nules Clementine	Hom	2	0	0	2
		Het	10	0	31	41
	Chandler pummelo	Hom	9	4	69	82
		Het	4	0	19	23
	Pink Pummelo	Hom	10	0	78	88
		Het	5	0	17	22
	Sweet Orange	Hom	-	-	0	0
		Het	-	-	72	72
	trifoliate orange	Hom	-	-	128	128
		Het	-	-	0	0
JoinMap Segregation type	Chandler x Nules	nnxnp	130	20	606	756
		lmxll	34	2	6	42
		hkxhk	1	0	29	30
		efxeg	43	3	0	46
		abxcd	70	0	0	70
	Nules x Pink	nnxnp	24	2	8	34
		lmxll	79	15	644	738
		hkxhk	3	1	24	28
		efxeg	19	5	0	24
		abxcd	26	0	0	26
	Orange x trifoliate orange	nnxnp	-	-	1	1
		lmxll	-	-	572	572
		hkxhk	-	-	9	9
		efxeg	-	-	0	0
		abxcd	-	-	0	0

**Figure 2:**
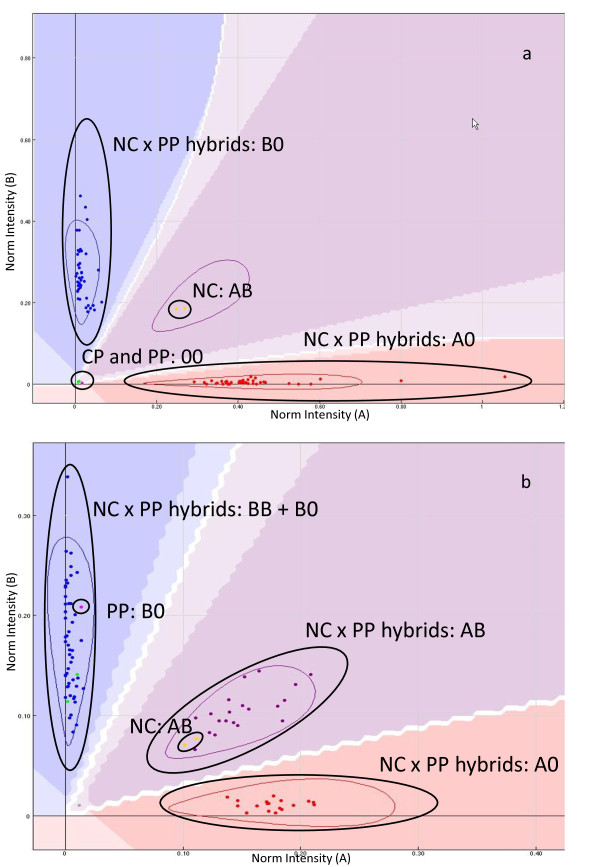
**Example of segregation profiles for SNP markers with null alleles for one parent and heterozygous for the other.** (**a**) AB × 00; (**b**) AB × B0.

The observed segregation within a progeny permitted identification of the null alleles in terms of homozygosity (00) or heterozygosity (A0) in the parents (Figure 2). These two configurations of null alleles were found for 0 and 31 markers in the Clementine, 69 and 19 in Chandler, 78 and 17 in Pink, 0 and 72 in sweet orange, and 128 and 0 in trifoliate orange, respectively (Table 2 and Additional file 1). Markers with A0 × BB and A0 × 00 configurations were treated as < lm × ll > and the reciprocal configurations were treated as < nn × np >. Markers with the AB × A0 configuration were analyzed as < lm × ll > by considering (i) BA and B0 hybrids as < lm > genotypes, (ii) the undistinguishable AA and A0 as < ll >; thus, considering only the segregation of the AB parent. Reciprocal configurations were treated as < nn × np >.

Considering all markers (with and without null alleles), the first category consisted of markers heterozygous in one parent and homozygous in the other (classified as < nn × np > or < lm × ll > in JoinMap). These markers represented the majority of the useful markers (with 606 < nn × np > and 6 < lm × ll > in CP × NC, 8 < nn × np > and 644 < lm × ll > in NC × PP and 1 < nn × np > and 572 < lm × ll > in SO × TO). These markers were only mapped for the heterozygous parents. As SNP markers are diallelic, the only other conformation encountered was < hk × hk >, where the two parents displayed the same heterozygosity. These markers were not frequent, and 29, 24 and 9 markers with such a configuration were observed for CP × NC, NC × PP and SO × TO, respectively. Considering our strategy to develop independent maps for each parent, the lack of information when assigning the parental allele for each hybrid (only possible for the homozygous hybrid and, thus, only half of the population) and the relatively low number of markers with this < hk × hk > conformation, these markers were removed from the mapping analysis.

### SSR and Indel genotyping

The genotyping of the CP × NC population was performed in the framework of the ICGC. SSR analysis was performed by six international groups (University of California at Riverside; University of Florida; University of Cukurova–Turkey; IVIA–Spain; INRA–France and CIRAD–France, with the collaboration of INRAM–Morocco). The genotyping of the NC × PP was performed at CIRAD and IVIA.

Homozygous or heterozygous null alleles in the parents were assumed from the observed SSR segregations. These two configurations of null alleles were found in 2 and 10 markers in Clementine, 9 and 4 in ‘Chandler’ and 10 and 5 in ‘Pink’, respectively (Table 2 and Additional file 1). Loci containing null alleles were treated as previously described for SNP markers. With multiallelic SSRs, six allelic configurations were possible. AA × AB or CC × AB were treated equally as < nn × np > by JoinMap, and the two reciprocal configurations were assumed to be < lm × ll >. Fully heterozygous configurations with four alleles (AB × CD) or three alleles (AB×BC) were coded < ab × cd > and < ef × eg >, respectively. Among the SSRs successfully genotyped, the five JoinMap configurations (nn × np, lm × ll, hk × hk, ef × eg, and ab × cd) were encountered for 130, 34, 1, 43 and 70 markers in CP × NC and 24, 79, 3, 19 and 26 markers in NC × PP progenies, respectively. As for SNPs, the very few markers with the hk × hk configuration were removed from the analysis. The nn × np and lm × ll markers were mapped for the male or female parents, respectively. The fully heterozygous markers (< ef × eg > and < ab × cd >) were mapped for the two parents and, therefore, allowed anchoring of the male and female parent maps.

Only four Indel markers displayed homozygous null alleles in ‘Chandler’ pummelo (Table 2 and Additional file 1). No heterozygous null alleles were indicated in ‘Nules’ Clementine, ‘Chandler’ or ‘Pink’ pummelos. For Indels, the five JoinMap configurations (nn × np, lm × ll, hk × hk, ef × eg, and ab × cd) were encountered for 20, 2, 0, 3 and 0 markers in CP × NC and for 2, 15, 1, 5, and 0 markers in NC × PP, respectively.

### Parental genetic mapping

Parental gamete genotypes were generated from the diploid data using nn × np, lm × ll, ef × eg and ab × cd scored markers. SNP, SSR and Indel genotyping data resulted in a matrix of 156 individuals and 872 markers for male Clementine (CP × NC progeny), 156 individuals and 158 markers for ‘Chandler’ pummelo (CP × NC progeny), 140 individuals and 788 markers for female Clementine (NC × PP progeny), 140 individuals and 84 markers for ‘Pink’ pummelo (NC × PP progeny), and 572 markers for 147 hybrids for sweet orange (SO × TO progeny). All of these matrices were analyzed using JoinMap 4. The linkage group numbering was performed according to the sweet orange genetic map established by the US citrus genome working group (Mikeal Roose; personal communication). The main results of the individual mapping analyses are given in Table 3, and detailed results are presented in Additional file 2.

**Table 3:** Main parameters of the six genetic maps inferred from three segregating progenies

	**N**	**LG 1**			**LG 2**			**LG3**			**LG 4**			**LG 5**		
		**M**	**D**	**Size**	**M**	**D**	**Size**	**M**	**D**	**Size**	**M**	**D**	**Size**	**M**	**D**	**Size**
Clementine F	140	96	3	118.08	92	9	120.06	137	2	159.42	85	13	66.13	108	36	108.34
Clementine M	156	98	54	131.09	110	4	155.69	160	88	208.00	95	68	114.17	124	103	124.30
Clementine F+ M	296	112	42	128.46	113	15	138.92	176	86	186.32	104	58	89.49	141	71	119.93
Chandler Pummelo	156	19	0	101.79	26	9	109.39	18	2	157.23	15	0	89.93	24	3	63.29
Pink Pummelo	140	8	0	67.29	10	1	100.37	4	0	39.34	6	2	69.07	15	0	71.11
Sweet Orange	147	54	13	71.70	27	1	54.33	117	25	93.15	64	2	76.22	96	48	99.87
	**N**	**LG 6**			**LG 7**			**LG 8**			**LG 9**			**Total**		
		**M**	**D**	**Size**	**M**	**D**	**Size**	**M**	**D**	**Size**	**M**	**D**	**Size**	**M**	**D**	**Size**
Clementine F	140	86	16	88.20	40	0	86.24	44	0	97.74	95	23	79.33	783	102	923.54
Clementine M	156	86	53	100.46	47	35	112.22	52	7	125.81	97	83	92.53	869	495	1164.26
Clementine F+ M	296	95	59	99.80	52	19	115.59	61	5	118.03	107	88	87.54	961	443	1084.07
Chandler Pummelo	156	19	0	64.83	8	0	53.96	16	6	115.17	6	0	73.03	151	20	828.62
Pink Pummelo	140	14	6	79.83	4	0	36.84	12	0	98.47	8	4	71.58	81	13	633.90
Sweet Orange	147	60	9	65.57	36	2	84.17	45	2	39.68	70	51	84.91	569	153	669.61

N: number of gametes; LG: linkage group; M: number of markers in the LG; D: number of markers with non-Mendelian segregation (p<0.05); Size: size of the LG in cM; F:female; M: male.

#### ‘Nules’ Clementine genetic map

The reference Clementine genetic map was obtained in two steps. In the first step, male and female Clementine data were analyzed separately.

Male Clementine map: Among the 872 segregating markers, 869 (606 SNPs, 240 SSRs and 23 Indels) were distributed into nine linkage groups (LGs) while three markers remained ungrouped. Most of the LG conserved their integrity until LOD=10. Only LG8 was disrupted in three sub-groups at LOD 9.The three sub-groups corresponded to three regions of LG8 separated by relatively wide intervals without intermediate markers. When mapped individually they displayed conserved order and very similar distances compared with the entire LG8. The map spanned 1164.26 cM. The Clementine male gametes exhibited 57% of the markers deviating from the expected Mendelian ratio (with a 0.05 probability threshold). Skewed markers were grouped within several parts of the genome. The skewed markers were unequally spread throughout the linkage groups with relatively low frequencies in LG2 (3.6%) and LG8 (13.5%), but with very high frequencies in LG4 (71.6%), LG5 (83.1%), LG7 (74.5%) and LG9 (85.6%). This distribution of segregation distortions is detailed below in comparison with the other parents.

Female Clementine map: Among the 788 markers successfully genotyped, 783 (642 SNPs, 122 SSRs and 21 Indels) were grouped in nine LGs, while five remained ungrouped. Most of the LG conserved their integrity until LOD=10. Only LG8 was disrupted in two sub-groups at LOD=8 corresponding to two regions of le LG8 separated by a relatively wide interval without marker. When mapped individually the sub-groups displayed conserved order and very similar distances compared with the entire LG8.The map size was 923.5 cM. The frequency of skewed markers (13.0%) was much lower than that observed among male gametes. Skewed markers were mainly concentrated in LG5 (33.3%) and LG9 (24.1%).

**Figure 3:**
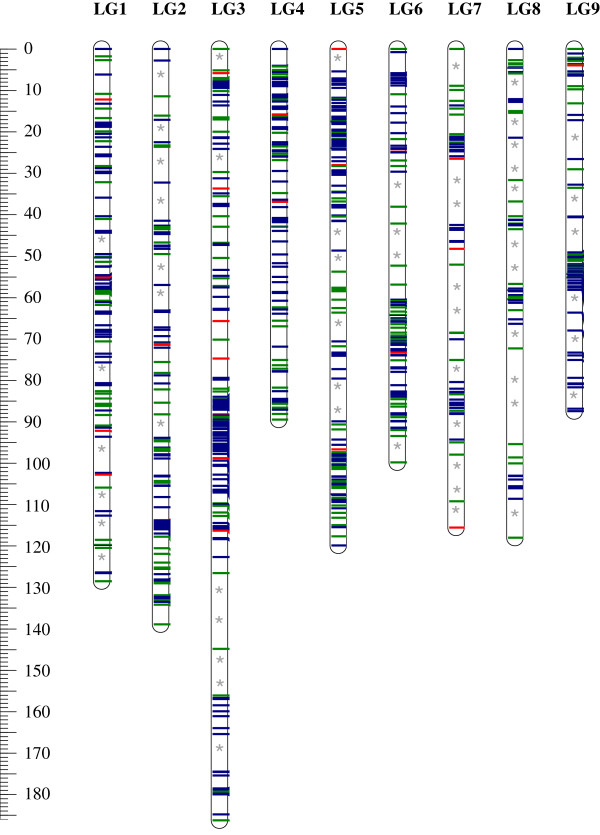
**Distribution of markers in the ‘Nules’ Clementine genetic map.** Red: Indels, green: SSRs, blue: SNPs, **interval between two markers > 10 cM; *interval between two markers > 5 cM and < 10 cM.

**Figure 4:**
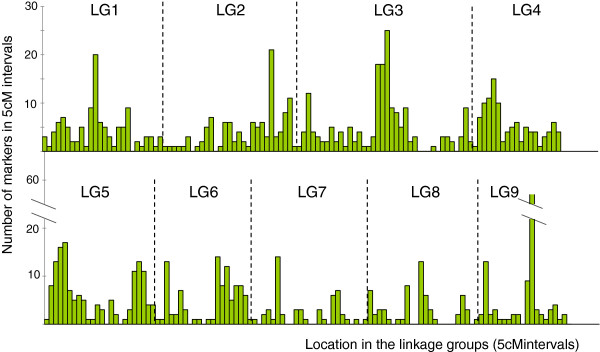
Density of markers along the ‘Nules’ Clementine genetic map.

Despite the high frequency of skewed markers in the male Clementine map, the colinearity between the male and female maps was highly conserved (Additional file 3). Therefore, the reference Clementine map was established by joining the two data sets for each LG, including all markers present in at least one map. Nine hundred and sixty-one markers (677 SNPs, 258 SSRs and 26 Indels) were grouped into nine linkage groups totaling 1084.07 cM (Figure 3 and Additional files 2 and 4). The proportion of skewed markers remained high (46.1% for p < 0.05). The LG size ranged from 87.5 cM (LG9) to 186.3 cM (LG3). LG7 and LG8 possessed a relatively low density of markers with an average of 0.45 and 0.52 markers/cM, respectively. On average, nearly one marker/cM was found on the other LGs. Each LG exhibited a heterogeneous density of markers (Figure 4). A few gaps larger than 10 cM were observed without mapped markers, and more gaps between 5 cM and 10 cM were observed without markers (Figure 3). These gaps were distributed, respectively, as follows: LG1 (0, 6), LG2 (0, 7), LG3 (2, 3), LG4 (0, 0), LG5 (1, 4), LG6 (1, 2), LG7 (3, 5), LG8 (3, 4) and LG9 (0, 6). On LG9, a special feature was observed, in which 55 markers were mapped within a 5-cM interval.

#### ‘Chandler’ pummelo genetic map

Among the 158 segregating markers, 151 (141 SSRs, 5 SNPs and 5 Indels) were successfully mapped in nine linkage groups (Additional files 2 and 5). One hundred and nine of these markers were common with the Clementine map. The level of segregation distortion was low (13.2%) and was mainly observed on two LGs (LG2: 34.6% and LG8: 37.5%). The total size of the map was 828.6 cM.

#### ‘Pink’ pummelo map

Only 84 segregating markers were available for Pink pummelo mapping. Eighty-one (67 SSRs, 7 SNPs and 7 Indels) were mapped in nine linkage groups (Additional files 2 and 6). Fifty-two of these markers were shared with the Clementine map. The level of segregation distortion was similar to the Chandler pummelo map (15.9%), but affected other LGs, mainly LG6 (42.9%) and LG9 (50%). The map spanned 633.9 cM.

#### Sweet orange map

The sweet orange map was only based on SNP markers. Among the 572 segregating markers, 569 were mapped in nine linkage groups, with a total size of 669.6 cM (Additional files 2 and 7). Most of the LG conserved their integrity until LOD=10. However three LG (2, 3 and 5) were disrupted in two sub-groups at LOD 9, 6 and 10 respectively. As for male and female clementine these disruptions corresponded to relatively wide interval without intermediate markers. When mapped individually the sub-groups displayed conserved order and very similar distances compared with their relative entire LGs. Four hundred and eighteen of these markers were in common with the reference Clementine genetic map. Segregation distortion was relatively frequent (26.9%) and was particularly clustered in LG5 (50%) and LG9 (72.9%).

### Genetic map comparisons

#### Analysis of colinearity between the different genetic maps

**Figure 5:**
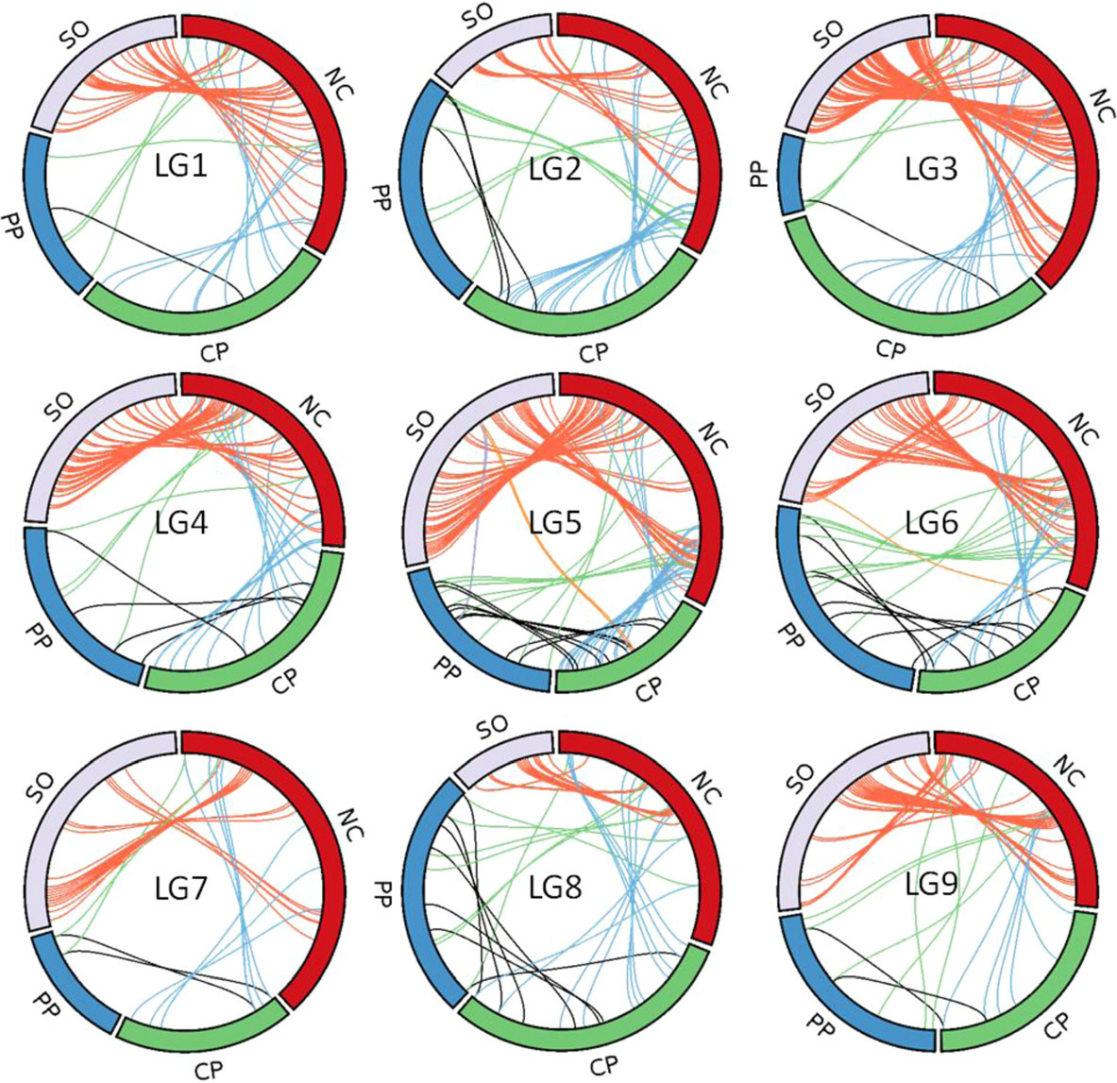
**Conservation of synteny and linear order of markers in the four genetic maps.** NC: ‘Nules’ Clementine, CP: ‘Chandler’ pummelo, PP: ‘Pink’ pummelo, SO: sweet orange.

Synteny, considered as the collocation of marker in the same chromosome, was completely conserved between all of the parental genetic maps. The linear order of the common markers was also highly conserved between parents (Figure 5), with only a few cases of inverted order in small intervals. However, the genetic distance between markers appeared to be unequal between parents. Sweet orange in particular displayed smaller distances between shared markers than Clementine. To avoid bias due to the different number of loci analyzed, new genetic maps of sweet orange and Clementine (male, female and consensus) were constructed using only the data generated from the 418 SNP markers that were successfully genotyped in the NC × PP, CP × NC and SO × TO progenies. The results (Additional file 8) confirmed that the genetic distances were generally lower (except for LG4 and LG9) in the sweet orange map than in the Clementine reference map. Moreover, differences were confirmed between the male and female Clementine maps for LG3, LG4, LG7, LG8 and LG9, with systematically lower distances in the female map. Interestingly, markers with very strong linkage localized in the very high marker density area of LG9 for the Clementine and sweet orange maps were much farther apart in ‘Chandler’ and ‘Pink’ pummelos (Figure 5).

#### Location of crossover events in the sweet orange gamete at the origin of Clementine and in the Clementine gamete at the origin of the haploid Clementine used for the reference citrus whole genome sequence

**Figure 6:**
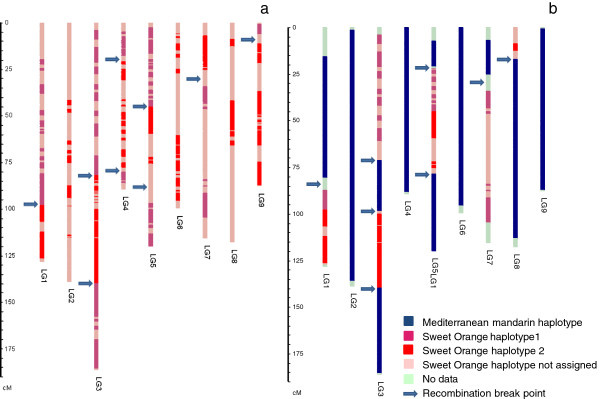
Haplotype constitution of the sweet orange gamete at the origin of Clementine (a) and of the haploid Clementine used to establish the reference whole citrus genome sequence (b).

For each linkage group, the haplotypes of sweet orange and Clementine were inferred from SNP marker phases given by JoinMap. The origin of Clementine from a ‘Mediterranean mandarin’ × sweet orange hybridization was proven by Ollitrault et al. [8]. Homozygous markers in sweet oranges and Mediterranean mandarin were used to identify the haplotype of Clementine inherited from sweet orange. Comparison of this haplotype with the two sweet orange haplotypes allowed the identification of nine recombination break points, one each in LG1, LG7 and LG9, and two each in LG3, LG4 and LG5 (Figure 6a). The two Clementine haplotypes were compared with the genotyping data of the haploid Clementine used by the ICGC to establish the reference citrus WGS haploid sequence. This permitted the identification of eight recombination break points, one each in LG1, LG7 and LG8, two in LG 5 and three in LG3 (Figure 6b). Interestingly, LG2, LG4, LG6 and LG9 appeared to have been entirely inherited from ’Mediterranean’ mandarin without recombination.

#### Comparative distribution of segregation distortions

To compare the location of the genome areas affected by segregation distortions in the different parental maps, a rough location in the reference Clementine maps was estimated for markers (i) mapped in sweet orange but not in Clementine, (ii) mapped in ‘Chandler’ pummelo but not in Clementine or sweet orange and, finally (iii) for markers only mapped in ‘Pink’ pummelo. These location estimates were performed by applying tendency curve equations of the location in the reference Clementine map (y axis) according to the location (x axis) for the parent map, where additional markers were mapped. An example of such a location is presented in Additional file 9b. The estimated locations of all markers in the framework of the Clementine reference map are given in the “synthesis” column of Additional file 2. The values of the X^2^ conformity test of the observed segregation against the 1:1 Mendelian hypothesis are represented along the linkage groups for all of the parental maps in Additional file 9a. Skewed markers appeared to be concentrated in specific areas for the different parents. However sporadic occurrences of a non-distorted marker within a cluster of distorted markers (CiC5563-02), or vice versa (e.g., marker CID5573) are observed in the Clementine reference map. Such exceptions can be explained by the inclusion of these markers with missing data, of probable non random origin, affecting the real segregation ratio.

The patterns of segregation distortion are consistent with the local selection of gametes that differ in terms of the probability of contributing to the next generation. Male Clementine presents the higher proportion of skewed loci. In LG1 and at the initial part of LG5, these distortions seem to be shared with female Clementine and sweet orange, although at a lower intensity than in male Clementine. Shared areas of skewed loci were also observed for male Clementine and sweet orange at the end of LG5 and in the middle of LG9, where high marker density was observed. In these two regions, the magnitude of sweet orange distortions was higher than in the male Clementine. The very severe level of segregation distortion observed in the middle of LG3 for male Clementine is shared at a much lower level with sweet orange. The skewed loci of male Pink pummelo in LG6 and LG9 were observed in areas common with male Clementine. Distortions that were observed in Chandler in the initial part of LG2 were not observed in the other parents.

**Figure 7:**
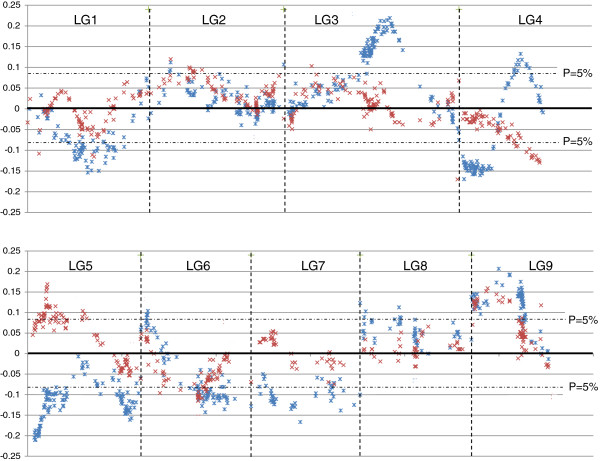
**Distribution of the segregation distortions for female and male Clementine, along the reference Clementine genetic map.** The x axis represents the location on each linkage group (LG) and y axis represents the excess of the mandarin allele relatively to Mendelian segregation (y = frequency of mandarin allele minus 0.5). Blue represents male Clementine segregation; red represents female Clementine segregation. The discontinuous lines represent the threshold for significant distortion (p < 0.05).

The identification of the Clementine haplotypes inherited from ‘Mediterranean mandarin’ and sweet orange allowed determining at each locus which allele was inherited from both parents of Clementine. Therefore, it was possible to determine which parental alleles (mandarin versus sweet orange) were favored for the skewed areas of the male and female Clementine segregations (Figure 7). No systematic tendency was observed. For male Clementine, the skewed segregations were globally in favor of sweet orange alleles for LG1, LG5 and LG7, while the skewed segregations favored mandarin alleles in LG3, LG8 and LG9. Interestingly, in LG6 and more markedly in LG4, a transition from positive selection for sweet orange alleles to positive selection for mandarin alleles was observed when moving from one end of the LG to the other. For LG1, LG2 and LG9, similar patterns of allele segregation were observed in female and male gametes (but generally with a lower distortion magnitude in the female). In LG4 and LG5, the patterns between male and female Clementine were very different, with significant distortion in opposite directions. In the second part of LG4, the mandarin alleles were favored in male Clementine, while sweet orange alleles were significantly favored in female Clementine. In the first part of LG5, mandarin and sweet orange allele were favored respectively in the female and male Clementine.

## Discussion

### A first reference genetic map for Citrus

The reviews of citrus genetic mapping performed by Ruiz and Asins [31], Chen et al. [19] and Roose [32] underlined that most of the earlier citrus genetic maps were based on intergeneric hybrids between *Citrus* and *Poncirus*. This was due to the importance of *Poncirus trifoliata* for rootstock breeding. Most of these studies suffered from relatively low numbers of analyzed hybrids and from the dominant nature of the markers (RAPD, AFLP) without sequence data on the mapped fragments. Several of the more recent maps were generated using co-dominant markers, particularly SSRs [17-19]. However, the number of mapped markers was insufficient to establish the nine linkage groups corresponding to the nine chromosomes present in haploid citrus. Some recent studies also focused on the genetic mapping of *Citrus* varieties [17,20,21,33]. The map of Gulsen et al. [21] was the first *C. clementina* map, while Bernet et al. [17] mapped Chandler pummelo and Fortune mandarin, a *C. clementina × C. tangerina* hybrid. None of these maps encompassed enough markers with published sequences to establish a reference citrus map useful to be combined with whole genome sequence data.

The current reference Clementine map, established from Clementine male and female segregation, includes 961 co-dominant markers (677 SNPs, 258 SSRs and 26 Indels) spread among nine LG. The map spans 1084.1 cM, with an average marker spacing of 1.13 cM. This is a substantially higher marker density than reported in previous citrus maps, in which nine LG were obtained. Omura et al. [34] established a genetic map spanning 801 cM with 120 CAPS markers. Sankar and Moore [35] published an 874 cM map including 310 markers (mostly ISSR and RAPD). Carlos de Oliveira et al. [20]) established an 845 cM map with 227 AFLP markers and more recently using 215 markers (mostly SRAP) Gulsen et al. [21] produced a 858 cM map.

The marker density in the current reference Clementine map varied along the genome. The density was particularly low in some regions of LG7 and LG8, with three gaps over 10 cM between markers in each of these LGs. The SNP markers are the most numerous markers on the Clementine map and were randomly selected. Therefore, these low marker density areas probably reveal highly homozygous regions of the Clementine genome. WGS data for the diploid Clementine will be very useful for developing targeted markers within these "no marker" regions. At the opposite extreme, high density areas were observed in some LGs. As described by Lindner et al. [36] and Van Os et al. [37], some of these high marker density regions may be associated with centromeric locations with large physical distances, possibly corresponding to low genetic distances. Another hypothesis is that some areas with high marker density correspond to portions of the genome in interspecific heterozygosity. Indeed, Clementine is considered to be a hybrid between Mediterranean mandarin and sweet orange [8,9,16]. As sweet orange is thought to have originated as a result of interspecific hybridization between *C. maxima* and *C. reticulata* gene pools [6,7,9], some parts of the Clementine genome may represent interspecific heterozygosity (*C. maxima/C. reticulata*). Garcia-Lor et al. [38] showed that the SNP/kb frequency was approximately six times higher between *C. reticulata* and *C. maxima* that it was within *C. reticulata.* Thus, randomly selected markers should be six times more frequent (by physical distance unit) in those parts of the Clementine genome involved in interspecific heterozygosity. Despite the heterogeneity of marker dispersion, the distance to the nearest mapped marker is less than 5 cM in most locations of the Clementine genome. Moreover previous published diversity studies done with the mapped SSRs (5, 23–26, 28), InDels (30) and SNPs (8) gave accurate information of their transferability and polymorphisms, at individual locus level, within and between the principal varietal groups. Therefore, this marker framework will be very useful for marker-trait association studies based on linkage disequilibrium, such as QTL analysis, bulk segregant analysis, or even genetic association studies in the mandarin group, where strong diversity was observed for the mapped SNP markers [8]. This map is being used to facilitate the chromosome assembly of the reference whole genome citrus sequence based on a haploid Clementine genotype [13,39].

### Linear marker order is highly conserved between species, but genetic distances are variable between sexes and species

The citrus genetic maps based on dominant and mainly cross-specific markers (such as RAPD, AFLP and ISSR) do not permit genetic map comparisons. Multi-allelic codominant markers, such as SSRs, are more powerful for such applications [30]. Chen et al. [19] and Bernet et al. [17] successfully used SSRs for citrus map comparison at the interspecific and intergeneric levels.

In the present study, the main genotyping effort concerned SNPs. Eight hundred and thirty-six SNP markers were genotyped in the three populations. Most of these markers were mined from Nules Clementine BAC end sequences [8,27] and, as a result, were heterozygous for Clementine. The development of the GoldenGate SNP markers from the Clementine sequence without information on the interspecific variability in flanking areas resulted in numerous homozygous null alleles in pummelo as described by Ollitrault et al. [8] and in trifoliate orange. Heterozygous null alleles for 72 markers were found in sweet orange, expanding the number of markers mapped in this species. The selected SNP markers were not efficient for pummelo or trifoliate orange mapping due to the very low number of heterozygous loci in these species. Moreover, the biallelic nature of SNP markers limited the establishment of two anchored maps (male and female) from a single cross. Therefore, comparison between Clementine and pummelo was still primarily limited to common multiallelic SSRs (109 between Clementine and Chandler pummelo and 52 between Clementine and Pink Pummelo). With sweet orange and Clementine maps being developed from different populations, the 418 common heterozygous SNPs allowed more substantial anchorage of the two maps.

The conservation of synteny was complete between the species, with no discrepancy in marker localization on the different linkage groups between the maps. Furthermore, the linear order of markers also appeared to be highly conserved between *C. clementina*, *C. sinensis* and *C. maxima*. This is in agreement with the conclusions of Bernet et al. [17] following their comparative study of partial maps between three species (*C. aurantium*, *C. maxima* and *P. trifoliata*) and Fortune mandarin, a Clementine-derived mandarin hybrid. In the present study, small localized inversions of marker orders were observed between maps, particularly in dense markers areas. Bernet et al. [17] concluded that similar results, for local ordering changes in the integrated maps, resulted from the inclusion of markers with missing data, and eventually different levels of distorted segregations between populations. It is also possible that small genotyping errors concerning the markers located in these dense regions disturbs the mapping order [40,41]. The fine mapping of such regions will require larger populations than the ones genotyped in this study. For this reason, these local inversions are not detailed in the results of this study since artifactual origins were quite probable. Chen et al. [19] also concluded that colinearity at the intergeneric level was highly conserved between genetic maps of *C. sinensis* and *P. trifoliata*. However, they also observed some inversions between shared loci that might reveal chromosomal rearrangement events, such as translocations or inversions. Considering the data of this study and the two previous comparative mapping studies, marker colinearity appears highly conserved at the intrageneric level (Clementine, mandarin, pummelo, sweet orange and sour orange), but also between *Citrus* and *Poncirus*. This global conservation of citrus genome organization will allow reasonable inferences of most citrus genome sequences via mapping NGS re-sequencing data to the haploid Clementine reference genome sequence.

Variations in LG sizes were observed between the current male Clementine and female Clementine maps. These variations were confirmed when the new maps were exclusively built using the markers shared between the three populations used for the implementation of the Clementine and sweet orange maps. Several LGs were longer in the male Clementine map than in the female one. This was observed in LGs with significant and extensive segregation distortions in the male haplotype populations compared with the female populations, and this was also observed in LG2, where very similar patterns of low skewed loci were observed. From simulated data, Hackett and Broadfoot [41] found that segregation distortion (due to gametic selection) alone had very little effect on marker order or map length. As discussed below, the observed distortion in Clementine probably results from gametic rather than zygotic selection. Therefore, it is probable that the longer LGs observed within the male Clementine map do not result from biased estimations due to segregation distortion, but instead reflect differential recombination rates. Such heterochiasmy between sexes is frequent in plants and animals [42-47]. According to species, recombination should be higher in male or in female gametes [43]. Despite the fact that heterochiasmy was documented early in the last century [44], there is still no consensus as to which of the several proposed hypotheses may explain its occurrence [45]. The various models were reviewed by Lenormand and Duteil [46]. Based on a large survey in animals and plants, these authors concluded that sexual heterochiasmy is not influenced by the presence of heteromorphic sex chromosomes; rather, it should result from a male–female difference in gametic selection. However, in this study, the citrus observations do not fit their global model considering as Trivers [47], that higher gametic selection in one sex reduced recombination in that sex to preserve the favorable gene combinations that confer reproductive success. Indeed, we found (see discussion on segregation distortion below) much more significant segregation distortion, and therefore probable gametic selection, for Clementine male gametes than for female gametes. The citrus data is more in agreement with models that suggest that the sex experiencing the more intense selection, or otherwise having the higher variance in reproductive success, should show more recombination (as reported by Burt et al. [47]).

Important differences in LG lengths were also observed between Clementine (male and female) and sweet orange for LG1, LG2, LG3, LG5, LG6 and LG8. The LGs for sweet orange were systematically shorter. The literature on plants and animals shows that the impact of structural heterozygosity on recombination frequency is variable. Different situations have been discussed by Parker et al. [48]. It is well established that sequence divergence at the interspecific level has an inhibitory effect on sexual recombination [49-52]. Chetelat et al. [52] observed a strong reduction in the recombination rate in a mapping population of an interspecific F1 tomato hybrid of *Lycopersicon esculentum* × *Solanum lycopersicoides*. The authors concluded that the high DNA sequence divergence between *L. esculentum* and *S. lycopersicoides* is a better explanation of reduced recombination than structural reorganization. Previously (and also in tomato), Liharska et al. [53] showed that the amount of recombination in a defined genetic interval decreased as the proportion of foreign chromatin (introgressed from close relatives of *L. esculentum*) increased. The authors also mentioned that, as the donor of the foreign chromatin became more distantly related, the level of observed recombination was lower. As the Clementine is a mandarin × sweet orange hybrid, and sweet orange arose from mandarin and pummelo gene pools (with a higher proportion of *C. reticulata*; [7,9]), it is highly probable that sweet orange contains more genome regions of interspecific heterozygosity (*C. reticulata*/*C. maxima*) than the Clementine. Therefore, it can be hypothesized that the lower LG sizes, and the associated lower recombination rates observed in sweet orange compared with Clementine, are associated with the relative interspecific patterns along the genome of these two species. The area of LG9 that displays substantially greater marker density in Clementine and sweet-orange suggests limited recombination within a large genome portion. Thus, two set of markers were common between the Clementine map and the two pummelo maps (MEST308, CIBE6092 and MEST065 for Pink pummelo and mCrCIR07F11, JI-AAG03, MEST 308 and CIBE6092 for Chandler pummelo). Interestingly, in the pummelo maps, these markers cover 26.5 cM and 30 cM, respectively, compared with an area concentrated within 2 cM in the Clementine map. It appears that both Clementine and sweet orange are strongly affected by a similar recombination limitation in LG9 for which they display equivalent map sizes. Haplotype analysis of sweet orange and diploid Clementine shows that the Clementine haplotype transmitted by sweet orange was inherited primarily from one of the sweet orange haplotypes, and only a small telomeric fragment was likely to be transmitted from the other sweet orange haplotype. Further genome analysis along with cytogenetic and mapping studies will be necessary to explain the different recombination patterns observed between species.

### Extensive segregation distortions are observed in specific linkage group areas particularly when Clementine is used as the male parent

Distortions from expected Mendelian allelic segregations were observed for all mapped parents of the segregating progenies. The highest rate was recorded for male Clementine with 56% skewed loci (p < 0.05). This percentage is more than four times higher than that of female Clementine (13%), which was equal with the estimate of female ‘Chandler’ pummelo. Male ‘Pink’ pummelo displayed a slightly higher level of distortion than female ‘Chandler’ pummelo (16%), while sweet orange (mainly from female data) displayed an intermediate level (27%). Distorted loci were also observed in most of the previous citrus mapping studies [17,20,54-57]. Bernet et al. [18] also reported a higher percentage of skewed loci in the male parents compared to the female parents in a reciprocal cross between ‘Chandler’ pummelo and ‘Fortune’ mandarin. Since most segregation distortions affect the allele frequencies without disturbing the genotypic frequency equilibrium (non significant F value–Wright fixation index; data not shown), it is probable that gametic selection was the main factor causing skewed segregation. Bernet et al. [17] reached the same conclusion from supporting biological data on parental fertility. Upon cross pollination with compatible parents, the proportion of fertilized ovules is much greater than the proportion of successful male gametes. Therefore, it appears logical that gametic selection is likely to be much more pronounced in male gametes than in females ones. This can result from several mechanisms such as gamete abortion, pollen competition or, the citrus gametophytic incompatibility system [58]. The pattern of X^2^ conformity test values, as well as the excess of mandarin alleles along the linkage groups, suggests that the presence of a small number of loci under relatively strong selection pressure on each chromosome is more likely than selection at multiple loci. Similar patterns were observed in tomato [52]. Identical areas of skewed loci were observed between Clementine and sweet orange in several linkage groups (LG1, LG3, LG5 and LG9). Modern sweet orange varieties arose from an interspecific hybrid prototype that has undergone vegetative propagation or propagation from seeds containing nucellar embryos over a several thousand year period. Besides favorable mutations and stable epigenetic variations that have been selected by man and the environment, it is probable that without the filter of sexual reproduction, the sweet orange genome accumulated unfavorable mutations in a heterozygous status. Some of these unfavorable mutations were likely transmitted to Clementine, as attested by the high proportion of weak progeny obtained from Clementine × sweet orange hybridization (our unpublished data), which should affect both sweet orange and Clementine segregations. Interestingly, the gametic selections have the same orientation for male and female Clementine in the genomic regions where sweet orange segregations are also skewed (LG1, end of LG5, and LG9). In other genome regions, male and female Clementine segregation distortions appeared disconnected. A very strong selection is observed in the middle of LG3 for the male Clementine, without significant skewing in the female. The male and female distortions appeared totally opposite at the end of LG4 and in the first part of LG5. The gametophytic incompatibility system described in citrus [58] could be a factor for male gametic selection. However, this may lead to a complete exclusion of one allele for the concerned locus and therefore, a very high distortion for the linked marker locus. This pattern was not observed in the present study. The gametophytic incompatibility system was also excluded as an explanation for the segregation distortion observed in the reciprocal crosses between ‘Fortune’ mandarin and ‘Chandler’ pummelo [17]. Some of the more extremely unequal allelic ratios (70/30) for the male Clementine occurred in areas without significant distortion (or even opposite selection) in the female. Such differences between male and female selection may partly explain the inconsistent results observed for trait segregation in the reciprocal crosses. Thus, it is difficult to infer genetic control from observed trait segregations without concomitant marker segregation analysis. This is particularly true if major genes controlling the studied trait are heterozygous in the male parent. QTL analysis may also be affected as described by Xu [59].

### Haplotype structure of the diploid Clementine and the haploid Clementine used for the implementation of the citrus whole genome reference sequence

Clementine is thought to have been selected as a chance seedling from a ‘Mediterranean’ mandarin by Father Clement just over one century ago in Algeria. The mandarin female parentage was confirmed by mitochondrial genome analysis [10]. The ‘Granito’ sour orange was initially considered to be the male parent [15]. However, molecular studies demonstrated that the Clementine was more likely a mandarin × sweet orange hybrid [8,9,16]. The marker phase analysis performed from the Clementine and sweet orange mapping data confirmed this hypothesis, and allowed the identification of the haplotype structures of the mandarin and sweet orange gametes that produced the Clementine. Nine recombination break points between the two sweet orange haplotypes (one each in LG1, LG7 and LG9, and two each in LG3, LG4 and LG5) were identified for the sweet orange gamete that produced the Clementine.

The implementation of a reference citrus whole genome sequence has been the primary focus of the ICGC for the last 5 years. Polymorphism in a whole genome sequence complicates the assembly process. Assembly contiguity and completeness is significantly lower than would have been expected in the absence of heterozygosity [60]. Commercial citrus varieties are characterized by high heterozygosity levels [6,7]. The comparison of blind versus "known-haplotype" assemblies of shotgun sequences obtained from a set of BAC clones from the heterozygous sweet orange [61] led the ICGC to establish the reference sequence of the citrus genome from a homozygous genotype. A haploid plant derived from the Clementine was selected due to its immediate availability and preexisting molecular resources [26,27,62-64]. The selected haploid was obtained by induced gynogenesis after *in situ* pollination with irradiated pollen [13]. The haploid Clementine was genotyped using the markers mapped in diploid Clementine and sweet orange. This permitted the constitution of the haploid genome to be determined according to the mandarin and sweet orange haplotypes constitutive of the diploid Clementine. Eight recombination break points were identified between the two Clementine haplotypes (one in LG1, LG7 and LG8; two in LG 5 and three in LG3). LG2, LG4, LG6 and LG9 appear to have been entirely inherited from the ’Mediterranean’ mandarin haplotype without recombination. Overall, a very large fraction of the genome of the haploid Clementine used for WGS was inherited from the ‘Mediterranean’ mandarin.

## Conclusions

Five parental genetic maps were established from three segregating populations that were genotyped using SNP, SSR and Indel markers. A first medium density reference map (961 markers for 1084.1cM) of citrus was established by joining male and female Clementine segregation data. Despite the heterogeneous dispersion of markers, this constitutes a good framework for further marker-trait association studies, and it has been used to enable the chromosome assembly of the reference whole genome citrus sequence [39]. The Clementine map was compared with two pummelo maps (‘Chandler’ map: 151 markers for 828.6 cM; ‘Pink’ map: 81 markers for 633 cM) and a sweet orange map (569 markers for 669.6 cM). The linear order of the markers appeared to be highly conserved at the interspecific level. This should allow for reasonable inferences of most citrus genome sequences via mapping NGS re-sequencing data in the haploid Clementine reference genome sequence. Important variations between the Clementine and sweet orange map sizes were observed, as well as variations between the male and female Clementine maps. This suggests variations in recombination rates. The smaller length of the sweet orange map is likely related to the higher interspecific heterozygosity within the sweet orange genome. Skewed segregations are numerous in the male Clementine map, underlining the potential extent of deviation from Mendelian segregation for characters controlled by heterozygous loci in the male parent. Genetic mapping data confirmed that the Clementine is a hybrid between the ‘Mediterranean’ mandarin and sweet orange. Nine recombination break points were identified between the two sweet orange haplotypes for the sweet orange gamete that contributed to the Clementine genome. The genome of the haploid Clementine used to establish the citrus reference sequence appears to be have been primarily inherited from the ‘Mediterranean’ mandarin haplotype of the diploid Clementine.

## Materials and methods

### Segregating progenies and DNA extraction

#### Clementine and pummelo genetic mapping

Two inter-specific segregating populations between *C. clementina* and *C. maxima* were used to establish the genetic maps. One hundred and fifty-six hybrids of ‘Chandler’ pummelo × ‘Nules’ Clementine (CP × NC) were produced and grown at CIRAD/INRA (Corsica), while 140 hybrids of ‘Nules’ Clementine × ‘Pink’ pummelo (NC × PP) were obtained at IVIA. Total DNA was extracted from fresh leaves according to Doyle and Doyle [65]. In addition to the interspecific hybrids, total DNA was extracted from the parental lines: diploid ‘Nules’ Clementine (IVIA-22), ‘Chandler’ pummelo (ICVN 0100608) and ‘Pink’ Pummelo (IVIA-275). DNA was also extracted from the haploid Clementine selected for the whole genome sequence implementation and ‘Mediterranean’ mandarin (IVIA-154), the assumed female parent of Clementine.

#### Sweet orange genetic mapping

One hundred and forty seven intergeneric hybrids between sweet orange and trifoliate orange (*Citrus sinensis × Poncirus trifoliata*; SO × TO) were used for sweet orange mapping using SNP markers shared with the Clementine map. These hybrids were obtained at UF-CREC (Florida) and previously used for sweet orange and trifoliate orange mapping using SSR markers [19]. The different crosses used were: (i) 56 hybrids of *C. sinensis* cv Sanford (Sa) × *P. trifoliata* cv Argentina (Ar), (ii) 40 hybrids of *C. sinensis* cv Fiwicke (Fi) × *P. trifoliata* cv Flying Dragon (FD); (iii) 15 hybrids of *C. sinensis* cv Ridge Pineapple (RP) × *P. trifoliata* cv Flying Dragon (FD), (iv) seven hybrids of *C. sinensis* cv Fiwicke (Fi) × *P. trifoliata* cv Argentina (Ar); (v) six hybrids of *C. sinensis* cv Ruby (Ru) × *P. trifoliata* cv Flying Dragon (FD), (vi) five hybrids of *C. sinensis* cv Ridge Pineapple (RP) × *P. trifoliata* cv DPI0906 (Ps), (vii) five hybrids of *C. sinensis* cv Ruby (Ru) × *P. trifoliata* Argentina cv (Ar), and (viii) 13 hybrids of *P. trifoliata* cv Flying Dragon (FD) × *C. sinensis* Ridge cv Pineapple (RP). Due to the nature of *C. sinensis* intraspecific evolution (somatic mutations but not sexual recombination), molecular polymorphisms between sweet orange cultivars is very rare [8,19]. Therefore, after confirming the lack of polymorphism between parental sweet oranges at the marker loci, all of the hybrids were considered to be derived from a single sweet orange genotype for the mapping analysis. Prior to DNA extraction, the ploidy level of all hybrids was estimated by flow cytometry, and only diploid hybrids were used. Genomic DNA was isolated from tender leaves using the CTAB method as described by Aldrich and Cullis [66].

### Markers

A total of 1166 markers were used to genotype the progenies. Of these markers, 837 were SNPs, 301 were SSRs and 28 were Indels.

#### SNPs

CiC****-**: the 802 SNPs were mined from the Clementine BAC end sequence database [27]. These markers are part of the 1536 total SNPs used to implement an Illumina GoldenGate assay. These markers were selected based on their quality and segregation in the analyzed progenies for at least one parent. They have been published by Ollitrault et al. [8] and the corresponding GenBank accession numbers can be found in Additional file 1.

ACO-*-***, ADC****, Aoc****, ATGGcM155, Cax4****, CHI-*-***, DXS-M-***, FLS-M-***; HKT1c800F141; LapXcF***; LCY2-*-***; LCYB-*-***, MDH-P-84; NADK2c800F***; PKF-M-186, PSY-M-289, TRPA-M-***, TScMI1331: These 34 SNP markers were mined by Sanger sequencing of 44 genotypes representative of *Citrus* and relative diversity, and were obtained from 19 genes implicated in the primary and secondary metabolite biosynthesis pathway and salt tolerance [38]. Corresponding GenBank accession numbers can be found in Additional file 1. Seventeen of these SNPs have been published [8]. Details on the 17 remaining markers can be found in Additional file 10.

#### SSR markers

The 301 SSR markers used for mapping were developed from genomic libraries (79), ESTs (188), and BACend sequences (34).

CI***** and mCrCIR*****: These 57 markers were developed by Froelicher and colleagues at CIRAD/INRA (France) from a genomic library of ‘Cleopatra’ mandarin. Corresponding GenBank accession numbers can be found in Additional file 1. Most of the mapped markers have been published [23,67-69]. Primers for the remaining markers are given in Additional file 11.

CIBE****: These 34 markers were developed by Ollitrault and colleagues at CIRAD/IVIA (France/Spain) from a Clementine BAC end sequence database [27]. These markers are published in Ollitrault et al. [28]. Corresponding GenBank accession numbers can be found in Additional file 1.

CF-*****, JI-***** and NB-****: These 59 markers were developed by Roose and colleagues at UCR (California). Fourteen of the markers are from genomic libraries and 45 are from ESTs. Corresponding GenBank accession numbers can be found in Additional file 1. Only the four NB-**** markers have been published [6]. Data on the remaining markers can be obtained upon request (Mikeal L. Roose <mikeal.roose@ucr.edu>).

CTV2745: This marker is closely linked to the citrus tristeza virus immunity gene of trifoliate orange and was developed in the Roose laboratory (UCR, California) from a genomic sequence [70].

Cms** and jk-****: These seven markers were developed from genomic libraries and were published by Ahmad et al. [71] and Kijas et al. [55], respectively.

CX****: These 70 markers were developed by Chunxian Chen and colleagues at the CREC (Florida) from an EST database. The corresponding GenBank accession numbers can be found in Additional file 1. Some of the mapped markers have been published by Chen et al. [19,25]. Data on the remaining markers can be obtained upon request (Chunxian Chen: cxchen@ufl.edu).

Mest****: These 73 markers were developed by Luro and Col. at INRA/CIRAD from EST databases (France). The corresponding GenBank accession numbers can be found in Additional file 1. Seven of these markers were published by Luro et al. [26]. The primer sequences of the remaining markers can be obtained upon request (luro@corse.inra.fr).

#### Indel markers

CID****: These 28 markers were developed from a Clementine BAC end sequence database [27] at IVIA/CIRAD (Spain), and have been published by Ollitrault et al. [30]. IDCAX is an Indel marker developed by Garcia-Lor et al. [7]. The corresponding GenBank accession numbers can be found in Additional file 1.

### Genotyping methods

#### SSRs

SSR genotyping was performed using different methods in different laboratories (Additional file 1).

At IVIA/CIRAD and INRA, PCR products (using wellRED oligonucleotides, Sigma®) were separated by capillary gel electrophoresis (CEQ™ 8000 Genetic Analysis System; Beckman Coulter Inc.) as described by Ollitrault et al. [28]. The data collection and analysis were performed with GenomeLab*™* GeXP software, version 10.0.

At CIRAD and Cukurova University, PCR products (using tailing M13 associated with three fluorescent dyes) were separated by electrophoresis on a Li-Cor DNA Analyzer 4200 system (Licor Biosciences, BadHomburg, Germany). The alleles were sized according to 50- to 350-bp standards (MWG Biotech AG, Ebersberg, Germany). SSR alleles were detected and scored using SAGA Generation 2 software (LI-COR, USA) and controlled visually.

At the CREC, PCR products (using tailing M13) were separated by capillary gel electrophoresis on an ABI 3130xl Genetic Analyzer (Applied Biosystems Inc., Foster City, CA, USA). GeneScan 3.7 NT and Genotyper 3.7 NT were used to extract the trace data and generate the microsatellite allele tables, respectively. More details can be found in Chen et al. [25].

At UCR, PCR products labeled by an M13-tailed primer strategy were separated using a denaturing 7% Long Ranger (BMA, Rockland, ME, USA) polyacrylamide gel attached to a LI-COR IR2 4200LR Global DNA sequencer dual dye system. Alleles were sized manually by comparison with 50–350 bp size standards (LI-COR), and then scored manually from gel image files. More details can be found in Barkley et al. [6].

#### Indels

Indel markers were genotyped by Capillary Gel Electrophoresis (CEQ™ 8000 Genetic Analysis System; Beckman Coulter Inc.) using wellRED oligonucleotides (Sigma®) as described by Ollitrault et al. [34]. Data collection and analysis were performed with GenomeLab*™* GeXP software, version 10.0.

#### SNPs

All SNP markers were genotyped on a GoldenGate array platform according to the standard Illumina GoldenGate assay instructions (http://www.illumina.com). More details can be found in Ollitrault et al. [8]. Two genotype controls (‘Nules’ Clementine and ‘Chandler’ pummelo) were repeated twice in each plate. The data were collected and analyzed using the Genome Studio software (Illumina). The automatic allele calling was visually checked for each marker/plate and corrected if necessary.

### Linkage analysis and genetic mapping

The two-way pseudo-testcross mapping strategy was used to determine the linkages in the different F1 populations from the two heterozygous parents as previously described [72] and used in previous mapping studies in citrus [17,19,73]. Each progeny was analyzed with JoinMap 4.0 [74]. The genotyping data were coded according to the “CP” population option adapted for such two-way pseudo-testcrosses with no previous knowledge of the marker linkage phases. In the first step, JoinMap was used to establish male and female gamete populations, which were analyzed separately. Segregation distortion was tested by χ2 conformity tests against the Mendelian segregation ratio of 1:1. Linkage analysis and marker grouping were performed using the independence LOD and a minimum threshold LOD=4. Phases (coupling and repulsion) of the linked marker loci were automatically detected by the software. Map distances were established in centiMorgans (cM) using the regression mapping algorithm and the Kosambi mapping function. Given that missing observations have much less negative impact on the quality of the map than errors, several authors recommend identifying suspicious data and treating them as missing observations [75,76]. In high density genetic mapping, a genotype error usually manifests itself as a singleton (or a double cross-over) under a reasonably accurate ordering of the markers. A singleton is a locus whose phase is different from both the marker phases immediately before and after. A reasonable strategy to deal with genotyping errors is to remove singletons by treating them as missing observations, and then refine the map by running the ordering algorithm [75,76]. For the Clementine map in which a relatively high number of markers was genotyped, singletons were automatically checked after a first mapping round and replaced by missing data using an excel page routine. The Clementine maps were established from these cleaned data. Distorted markers were not removed from the analysis because they were very frequent for some parents. Moreover, using JoinMap, each grouping of linked loci was based upon a test for independence in a contingency table. Since the test for independence is not affected by segregation distortion like the LOD score used by other methods of linkage analysis, a lower incidence of spurious linkage is expected [74]. The linkage maps were drawn using the MapChart program [77]. The circle plot diagram used to compare the marker order in four genetic maps was performed using Circos software (http://circos.ca/). Clementine and sweet orange haplotypes were drawn with GGT 2.0 software [78].

## Competing interests

The authors declare that they have no competing interests.

## Authors’ contributions

PO managed the work, analyzed the data and wrote the manuscript. JT and MT provided the SNP markers and contributed to data analysis. DB, ABe, ABo and AC developed the GoldenGate array and performed the SNP genotyping. YF developed the CP×NC population and provided DNA. PA developed the CN×PP population and provided DNA. CC and FGG provided the SO×TO progeny DNA and performed part of the SSR genotyping. CTF, SL, IH, FO, GC, YK, LM, AGL, CB, LN, FL and MLR contributed to the SSR and Indels genotyping, and JC contributed to the analysis of mapping data. All authors have read and approved the final manuscript.

## Supplementary Material

Additional file 1 **Origin and information for all markers.** This file contains a table showing detailed information for all markers: type of marker (Indels, SSRs or SNPs); the type of sequence data from which the markers were developed (genomic library, BAC end sequences, ESTs); GenBank accession number; the laboratory in which the markers were developed; the laboratory in which the different progenies were genotyped, the occurrence and configuration of null allele for the parents of analyzed progenies and the references for the papers in which the markers were published, with an indication of the modifications (if any) in the marker names. Click here for file

Additional file 2 **Detailed results of genetic mapping.** This file contains the detailed information (marker locations, X^2^ for Mendelian segregation, and level of significance) on the genetic maps for male Clementine, female Clementine, reference Clementine, sweet orange, ‘Chandler’ pummelo and ‘Pink’ pummelo. The estimated location of all markers in the reference Clementine map is also provided (synthesis columns). Click here for file

Additional file 3 **Conserved linear order between male and female Clementine genetic maps.** This file contains a figure showing the relative positions of the markers in the female Clementine map (y axis) and in the male Clementine map (x axis) for each linkage group. Click here for file

Additional file 4 **Reference Clementine genetic map.** This file contains a figure showing the nine linkage groups of the reference Clementine genetic map and the position of each marker (blue: SNPs; green: SSRs; red: Indels). Click here for file

Additional file 5 **‘Chandler’ pummelo genetic map.** This file contains a figure showing the nine linkage groups from the ‘Chandler’ pummelo genetic map and the position of each marker (blue: SNPs; green: SSRs; red: Indels). Click here for file

Additional file 6 **‘Pink’ pummelo genetic map.** This file contains a figure showing the nine linkage groups of the ‘Pink’ pummelo genetic map and the position of each marker (blue: SNPs; green: SSRs; red: Indels). Click here for file

Additional file 7 **Sweet orange genetic map.** This file contains a figure showing the nine linkage groups of the sweet orange genetic map and the position of each marker (blue: SNPs). Click here for file

Additional file 8 **Variation of map length between male Clementine, female Clementine, and sweet orange based only on common SNP markers.** This file contains a figure for each linkage group showing the relative position of the markers in the female Clementine map, the male Clementine map, and the sweet orange map in a new mapping analysis performed using only the common markers for the three parents. The x axis represent the location on the reference Clementine map established from all Clementine gametes (male + female). The relative locations in the other maps (the ratio between the locations in the other map relative to the location in the Clementine reference map) are shown on the y axis. Click here for file

Additional file 9 **Comparative distribution of the skewed markers in the nine linkage groups for five parents.** This file contains a figure for each linkage group showing the distortion magnitude (X^2^ of conformity with Mendelian segregation) for each marker and each mapped parent. Furthermore, 9b shows an example illustrating the method used to estimate the location in the reference Clementine map of markers mapped in the other parents. Click here for file

Additional file 10 **Information on the new SNP markers included in the GoldenGate array.** This file contains information regarding the new SNP markers included in the GoldenGate array. It includes the GenBank accession number, the sequence surrounding the SNPs, SNP position, the GoldenGate primers and designability rank. Click here for file

Additional file 11 **Characteristics and primers for the new SSR markers developed from ‘Cleopatra’ mandarin genomic library at CIRAD.** This file contains information on the primers used for the new SSRs developed from a Cleopatra mandarin (*C. reshni*) genomic library (GenBank accession number, primer sequences, annealing temperature and microsatellite motif). Click here for file
