# Supplementary material for: A reference genetic map of C. clementina hort. ex Tan.; citrus evolution inferences from comparative mapping
Source: BMC Genomics. 2012 Nov 5;13:593. doi: 10.1186/1471-2164-13-593 (PMC3546309; doi:10.1186/1471-2164-13-593)
Supplement: Additional file 3 — Conserved linear order between male and female Clementine genetic maps. This file contains a figure showing the relative positions of the markers in the female Clementine map (y axis) and in the male Clementine map (x axis) for each linkage group. [file 1471-2164-13-593-S3.pdf]

Ollitrault et al. (2012) A reference genetic map of C. clementina hort. ex Tan.; citrus evolution inferences from comparative mapping
BMC Genomics.2012, 13:593.

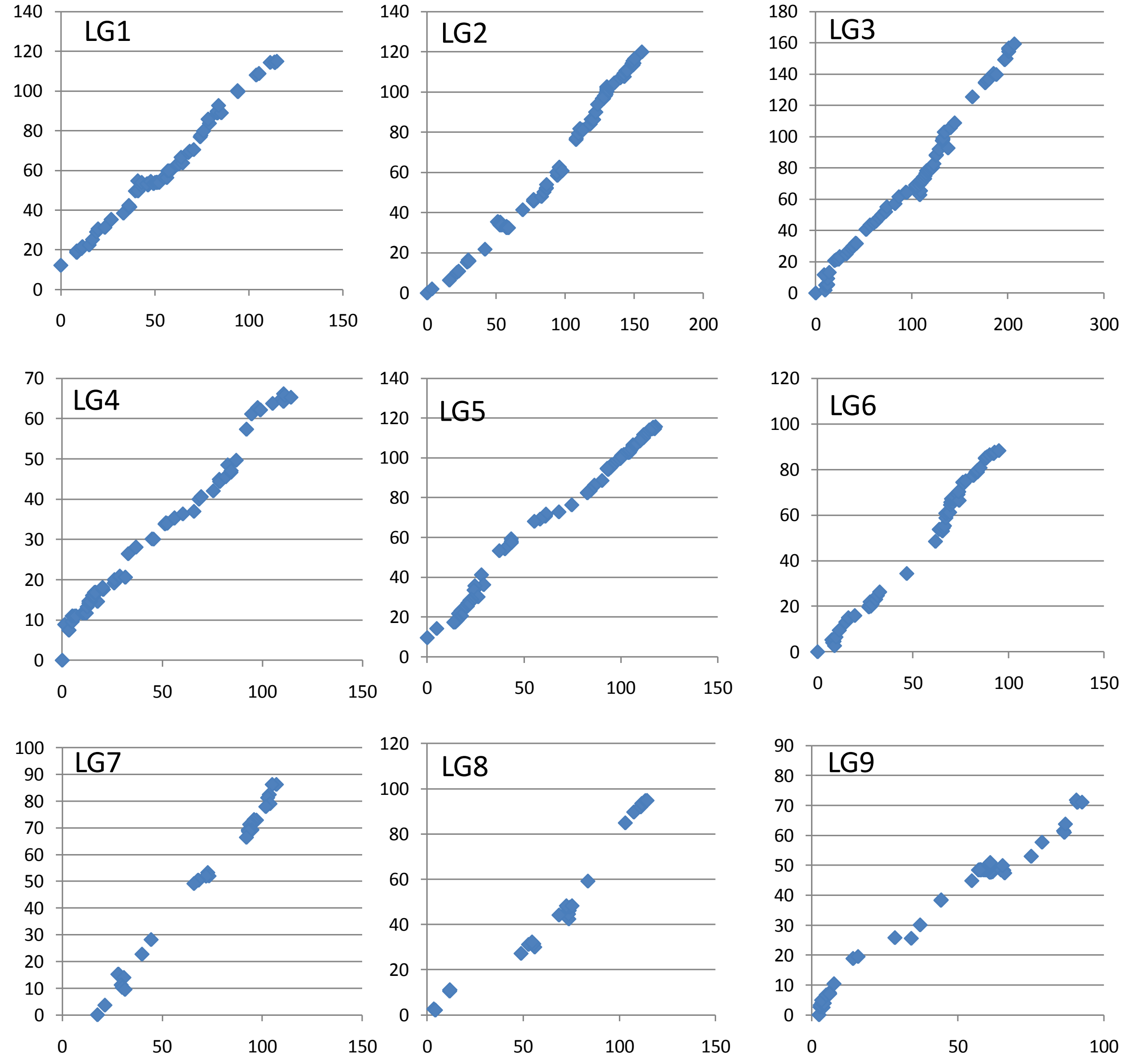


**Additional File 3:** Conserved linear order between male and female Clementine genetic maps

(x axis male map location in cM; y axis female map location in cM)
