# Supplementary figures and images for: A reference genetic map of C. clementina hort. ex Tan.; citrus evolution inferences from comparative mapping
Source: BMC Genomics. 2012 Nov 5;13:593. doi: 10.1186/1471-2164-13-593 (PMC3546309; doi:10.1186/1471-2164-13-593)

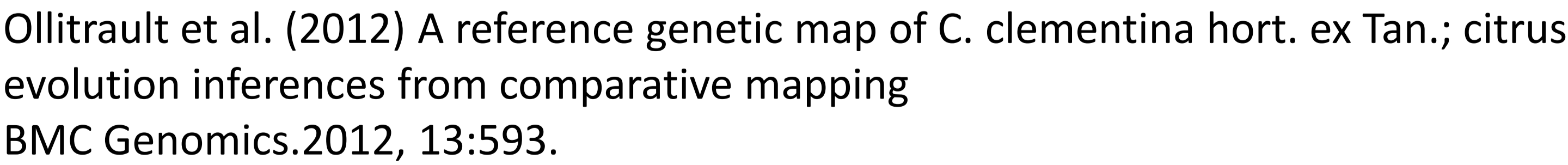

Supplement: Additional file 4 — Reference Clementine genetic map. This file contains a figure showing the nine linkage groups of the reference Clementine genetic map and the position of each marker (blue: SNPs; green: SSRs; red: Indels). [file 1471-2164-13-593-S4.pdf]

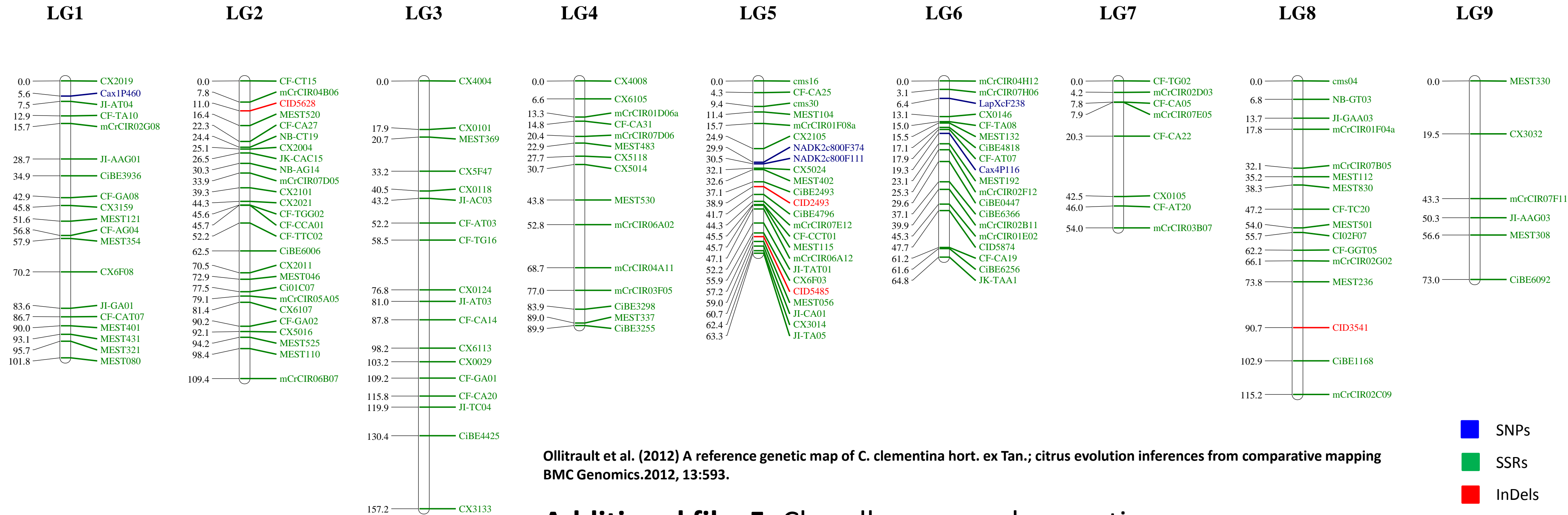

Supplement: Additional file 5 — ‘Chandler’ pummelo genetic map. This file contains a figure showing the nine linkage groups from the ‘Chandler’ pummelo genetic map and the position of each marker (blue: SNPs; green: SSRs; red: Indels). [file 1471-2164-13-593-S5.pdf]

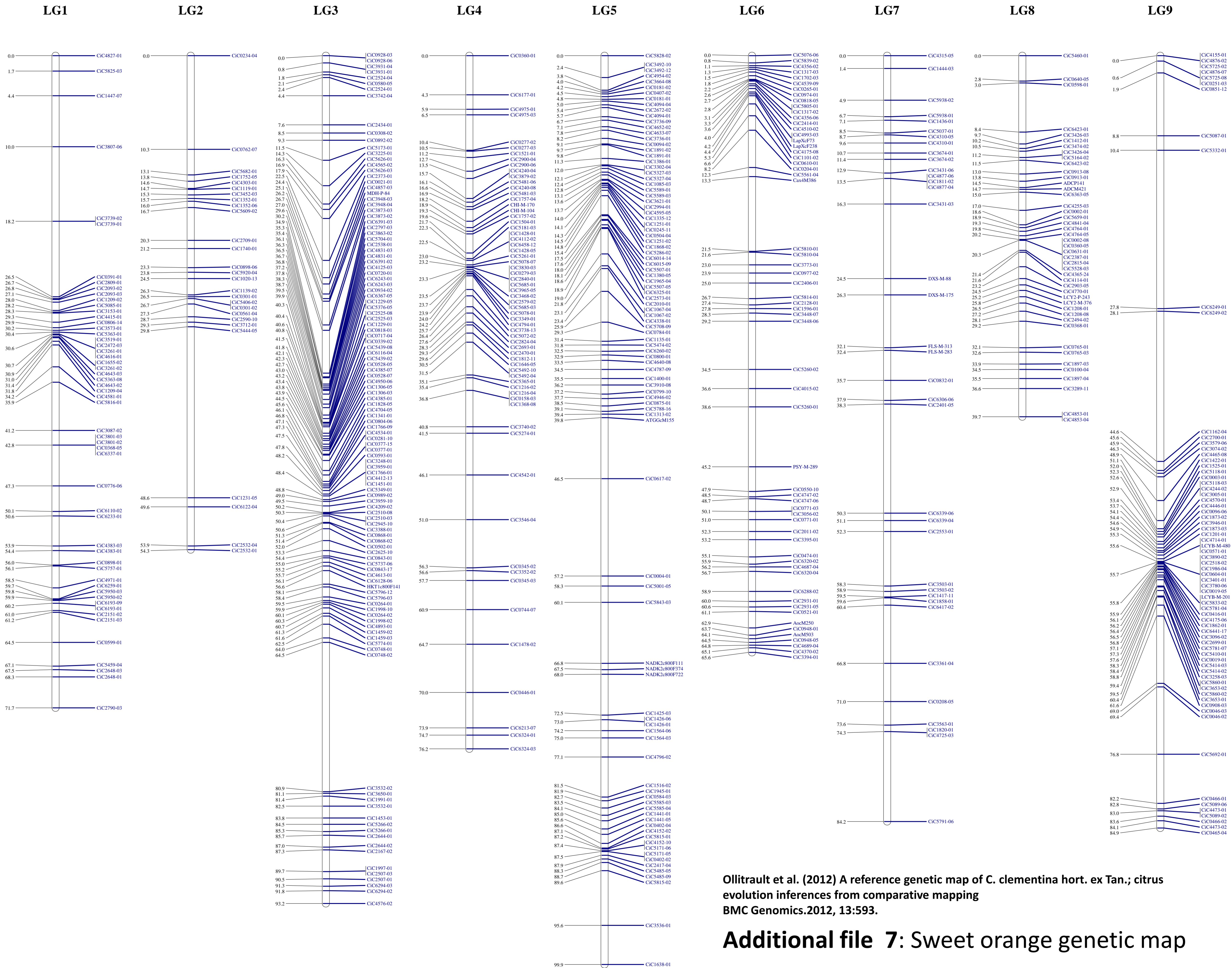

Supplement: Additional file 7 — Sweet orange genetic map. This file contains a figure showing the nine linkage groups of the sweet orange genetic map and the position of each marker (blue: SNPs). [file 1471-2164-13-593-S7.pdf]
