# Supplementary material for: A reference genetic map of C. clementina hort. ex Tan.; citrus evolution inferences from comparative mapping
Source: BMC Genomics. 2012 Nov 5;13:593. doi: 10.1186/1471-2164-13-593 (PMC3546309; doi:10.1186/1471-2164-13-593)
Supplement: Additional file 6 — ‘Pink’ pummelo genetic map. This file contains a figure showing the nine linkage groups of the ‘Pink’ pummelo genetic map and the position of each marker (blue: SNPs; green: SSRs; red: Indels). [file 1471-2164-13-593-S6.pdf]

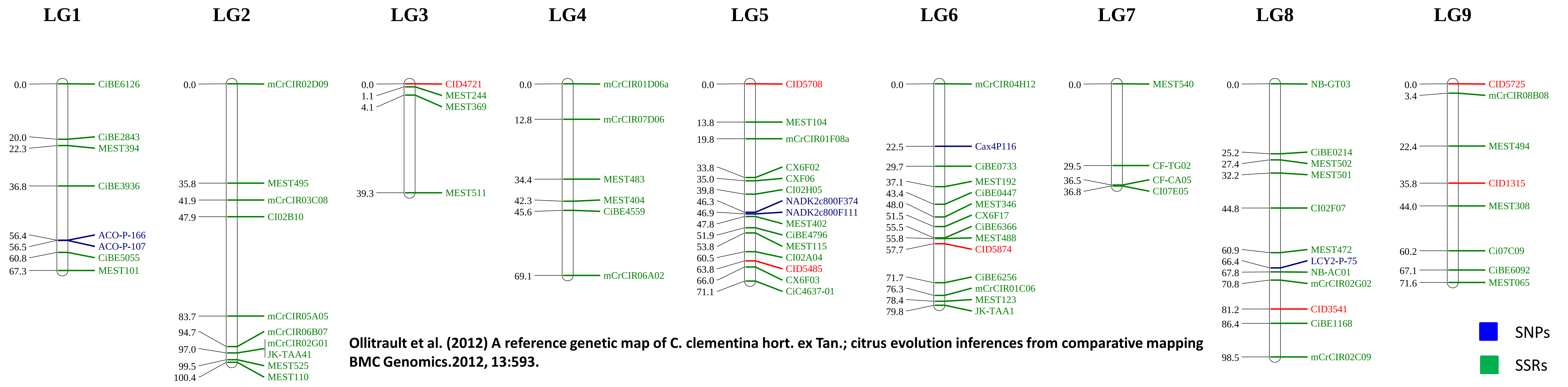

Ollitrault et al. (2012) A reference genetic map of C. clementina hort. ex Tan.; citrus evolution inferences from comparative mapping BMC Genomics.2012, 13:593.

Additional file 6: Pink pummelo genetic map
