# Supplementary material for: A reference genetic map of C. clementina hort. ex Tan.; citrus evolution inferences from comparative mapping
Source: BMC Genomics. 2012 Nov 5;13:593. doi: 10.1186/1471-2164-13-593 (PMC3546309; doi:10.1186/1471-2164-13-593)
Supplement: Additional file 8 — Variation of map length between male Clementine, female Clementine, and sweet orange based only on common SNP markers. This file contains a figure for each linkage group showing the relative position of the markers in the female Clementine map, the male Clementine map, and the sweet orange map in a new mapping analysis performed using only the common markers for the three parents. The x axis represent the location on the reference Clementine map established from all Clementine gametes (male + female). The relative locations in the other maps (the ratio between the locations in the other map relative to the location in the Clementine reference map) are shown on the y axis. [file 1471-2164-13-593-S8.pdf]

Ollitrault et al. (2012) A reference genetic map of C. clementina hort. ex Tan.;
citrus evolution inferences from comparative mapping
BMC Genomics.2012, 13:593

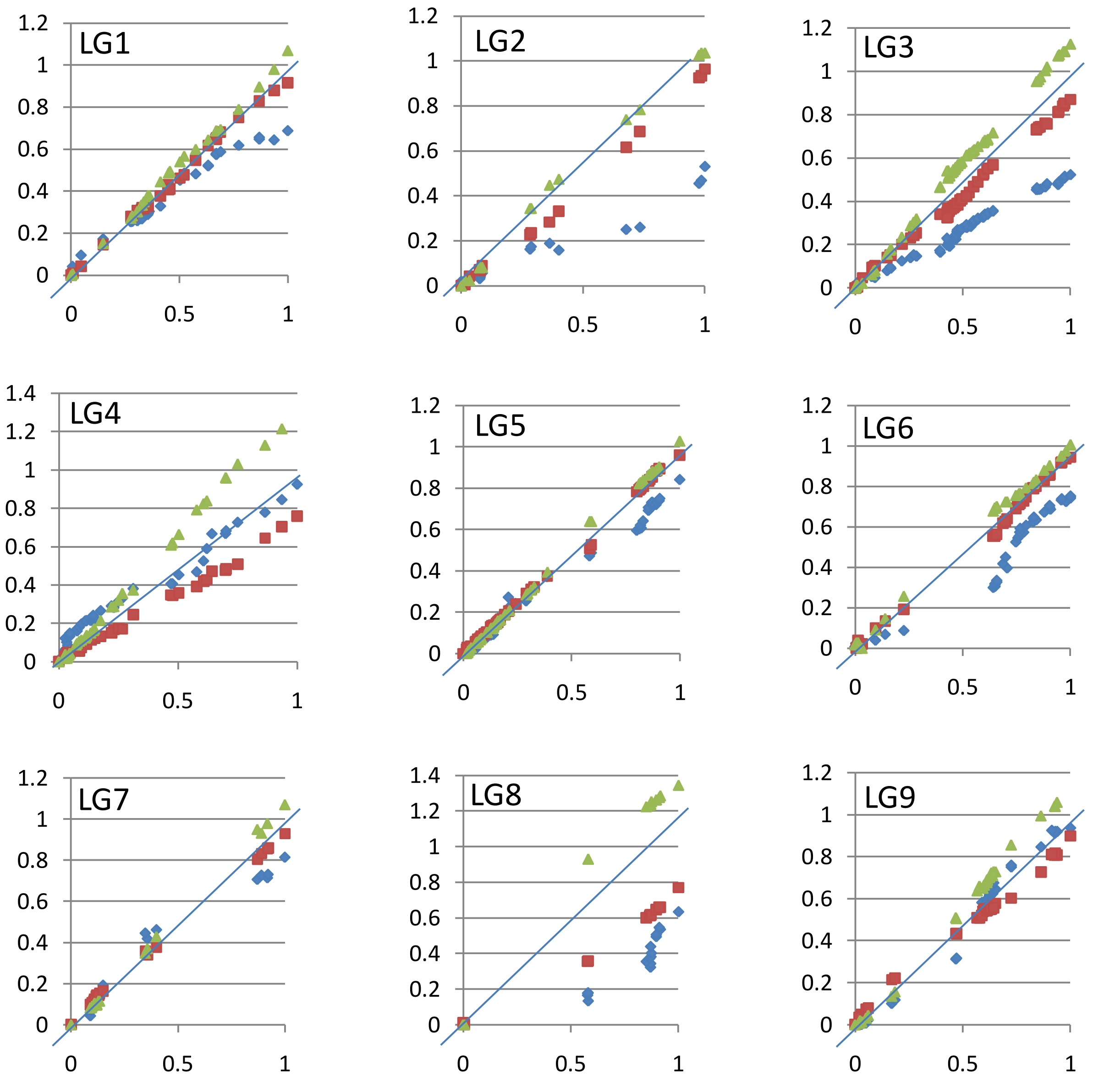

Additional file 8: variation of map length between male clementine, female clementine and sweet orange based only on the common SNP markers.

The location on the reference clementine map established from all clementine gametes (male + female) is used as x axis. Y axis are the relative location in the other maps (ratio between the location in the other map over the location in the clementine reference map). The diagonal blue line represent this reference clementine map (y=x). Green: male clementine map, red: female clementine map and blue: sweet orange map.
