# Supplementary material for: A reference genetic map of C. clementina hort. ex Tan.; citrus evolution inferences from comparative mapping
Source: BMC Genomics. 2012 Nov 5;13:593. doi: 10.1186/1471-2164-13-593 (PMC3546309; doi:10.1186/1471-2164-13-593)
Supplement: Additional file 9 — Comparative distribution of the skewed markers in the nine linkage groups for five parents. This file contains a figure for each linkage group showing the distortion magnitude (X2 of conformity with Mendelian segregation) for each marker and each mapped parent. Furthermore, 9b shows an example illustrating the method used to estimate the location in the reference Clementine map of markers mapped in the other parents. [file 1471-2164-13-593-S9.pdf]

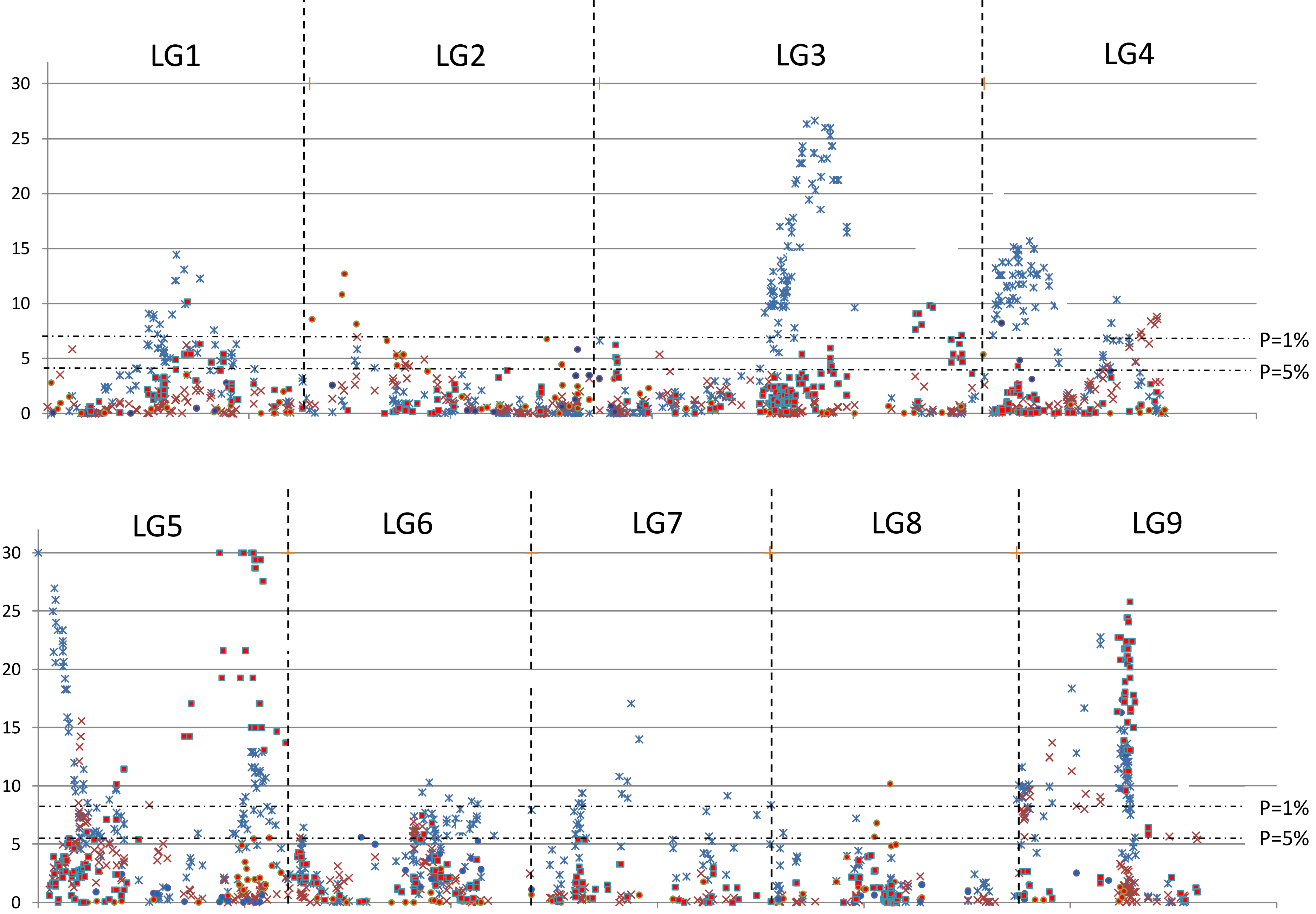

Ollitrault et al. (2012) A reference genetic map of C. clementina hort. ex Tan.; citrus evolution inferences from comparative mapping. BMC Genomics.2012, 13:593

Additional file 9: Comparative distribution of the skewed markers for the different parents in the nine linkage groups

X axis is the location of the markers on the reference clementine map; for markers mapped on sweet orange and pummelo but not present in the clementine map, the position was estimated by using the best tendencial curve equation (these locations are given in the synthesis column of additional file 1 ; the sequencial location of the markers not mapped in clementine was done as follow: first additional markers in sweet orange, 2nd additional markers in Chandler and 3rd: remaining markers in Pink.)
Y axis are the value of the conformity X^2 test against 1/1 segregation
Blue cross: male Nules clementine ; red cross: female Nules clementine; red circle: Chandler pummelo; blue circle: Pink Pummelo; red square : sweet orange
P: threshold for the X^2 test for probability 0.05 and 0.01

Additional file 9b: Estimation of the location of markers in the clementine reference map from locations in other maps.

A rough location in the reference clementine maps was estimated for markers (i) mapped in sweet orange but not in clementine, (ii) then for the markers mapped in Chandler pummelo but not in clementine nore in sweet orange and by the end (iii) for the markers only mapped in Pink pummelo. These location estimations were performed applying the equations of the tendencies curves of the location in the reference clementine map (y axis) according to the location (x axis) for the parent map where additional markers were mapped.

An example is given below for the linkage group 1.

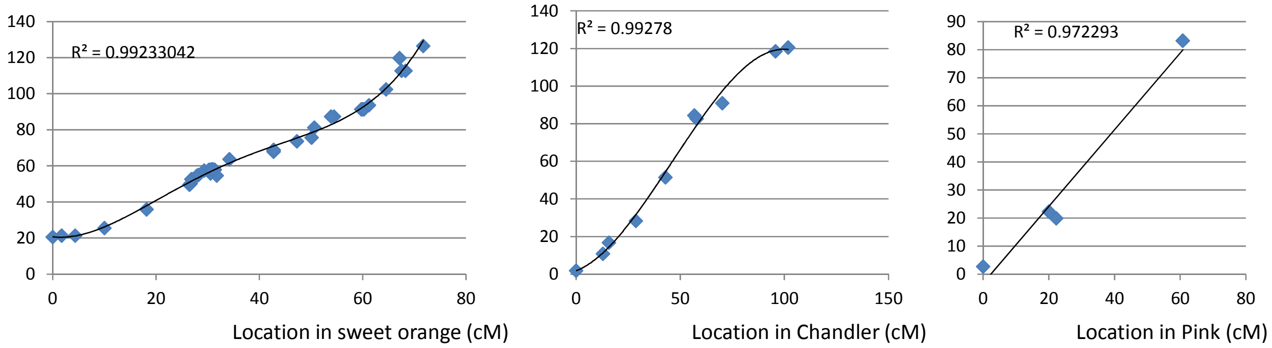

Tendencial curves established from shared markers between the reference clementine map (y axis) and the other genotypes in LG1

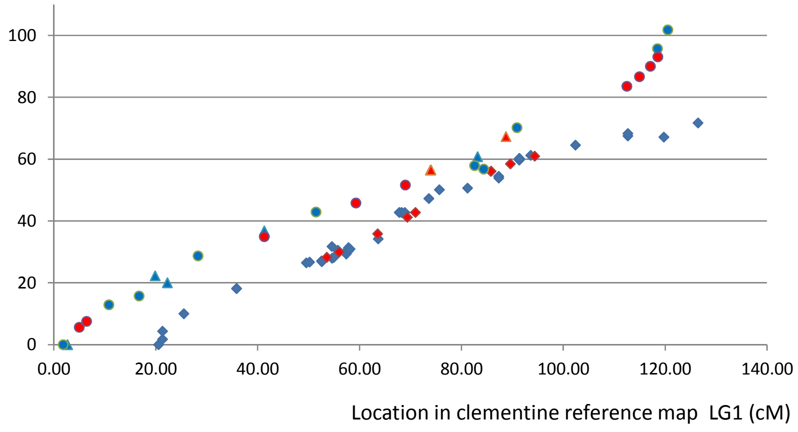

Red symbol represent the estimated positions in the reference clementine map (x axis) according to the locations in the sweet orange map (losange), in the Chandler map (circle) and in the Pink map (triangle) given in the y axis. Bue symbols are the relative locations of the shared markers for the sames maps.

The estimated position in the clementine reference map are given with the one established from clementine segregation in the column “Synthesis” of the additional file 2.
