# Supplementary material for: A reference genetic map of C. clementina hort. ex Tan.; citrus evolution inferences from comparative mapping
Source: BMC Genomics. 2012 Nov 5;13:593. doi: 10.1186/1471-2164-13-593 (PMC3546309; doi:10.1186/1471-2164-13-593)
Supplement: Additional file 11 — Characteristics and primers for the new SSR markers developed from ‘Cleopatra’ mandarin genomic library at CIRAD. This file contains information on the primers used for the new SSRs developed from a Cleopatra mandarin (C. reshni) genomic library (GenBank accession number, primer sequences, annealing temperature and microsatellite motif). [file 1471-2164-13-593-S11.pdf]

Additional file 11: Characteristics and primers of the new SSR markers developed from genomic 'Cleopatra' mandarin library at Cirad

Marker	GeneBank	motif	Foward	Reverse	T°m
mCrCIR01D10	FR692357	(GA)9	AAGCGACGGAAATAGAGAG	ATGGGGATGGGTGATGAAT	50
mCrCIR02B11	FR692358	(GA)11	GTATTTGGCGTGATGAA	CAAAGTAAATAGGGTGTGAG	50
mCrCIR02C09	FR692359	(GT)3(GA)9	TACTGACTGACCCCACC	TCCCCGTCTCCTACC	50
mCrCIR02D03	FR692360	(GA)8CA(GA)4X4(GA)8	CAGACAACAGAAAACCAA	GACCATTTTCCACTCAA	50
mCrCIR02E08	FR692361	(TGA)4(TCA)5	GGTTTGTGGGAGGTG	TGATTAGCATGTTGCG	50
mCrCIR02G08	FR692362	(GA)10	CATGCAATGTTCCACTT	AGGCAGTTGTTAGACCC	50
mCrCIR02H05	HE801214	(GA)13	GCATCATCCTACTTCTGTT	TGGAGGACTTGTGATTG	50
mCrCIR03E06	FR692363	(GT)8	AATACACCCTTCAAATCC	CTCCTAACAGATTTCATTACTC	50
mCrCIR03F05	FR692364	(GT)12	CTAAGGAAGAGTAGAGAGCA	TAAAATCCAAGGTTCCA	50
mCrCIR04A02	FR692365	(GA)11	GTTGTTGGTGTTGGTGT	TTCTCTCTGGTGGTGG	50
mCrCIR04A11	FR692366	(GT)12	GCACTGTAACAAACAACA	AATCCAAGGTTCCAAA	50
mCrCIR04B06	FR692367	(AT)4(GT)13	TTTTGTGTGAATGTTGG	GGAAATATCTTACTTGTGCT	50
mCrCIR04F01	FR692368	(GA)12	TCTTGTGAATGTTAGGCA	ACTTACGACACAAAACACAC	50
mCrCIR04F12	FR692369	(GAGT)3(GT)7	AAACAATCTTACAAGCCAC	TGTCCTGGGTTTTACTTAC	50
mCrCIR04H09	FR692370	(GT)11	GGGTCTGGATTTTGATT	CCATTTAGTGCCCAAG	50
mCrCIR04H12	FR692371	(CAT)7	TTCCTCTACAACTACAACCA	ATTATCCTCAACCTCCAA	50
mCrCIR05A04	FR692372	(GA)8	AAACGAGACAAGACCAAC	TATCAAACTCCCCTCACT	50
